# Machine learning unifies flexibility and efficiency of spinodal structure generation for stochastic biomaterial design

**DOI:** 10.1038/s41598-023-31677-7

**Published:** 2023-04-03

**Authors:** Zhuo Wang, Rana Dabaja, Lei Chen, Mihaela Banu

**Affiliations:** 1 Department of Mechanical Engineering, University of Michigan, Ann Arbor, MI 48109 USA; 2 Department of Mechanical Engineering, University of Michigan-Dearborn, Dearborn, MI 48128 USA

**Keywords:** Implants, Computational methods, Computational science, Mechanical engineering

## Abstract

Porous biomaterials design for bone repair is still largely limited to regular structures (e.g. rod-based lattices), due to their easy parameterization and high controllability. The capability of designing stochastic structure can redefine the boundary of our explorable structure–property space for synthesizing next-generation biomaterials. We hereby propose a convolutional neural network (CNN) approach for efficient generation and design of spinodal structure—an intriguing structure with stochastic yet interconnected, smooth, and constant pore channel conducive to bio-transport. Our CNN-based approach simultaneously possesses the tremendous flexibility of physics-based model in generating various spinodal structures (e.g. periodic, anisotropic, gradient, and arbitrarily large ones) and comparable computational efficiency to mathematical approximation model. We thus successfully design spinodal bone structures with target anisotropic elasticity via high-throughput screening, and directly generate large spinodal orthopedic implants with desired gradient porosity. This work significantly advances stochastic biomaterials development by offering an optimal solution to spinodal structure generation and design.

## Introduction

**Figure 1:**
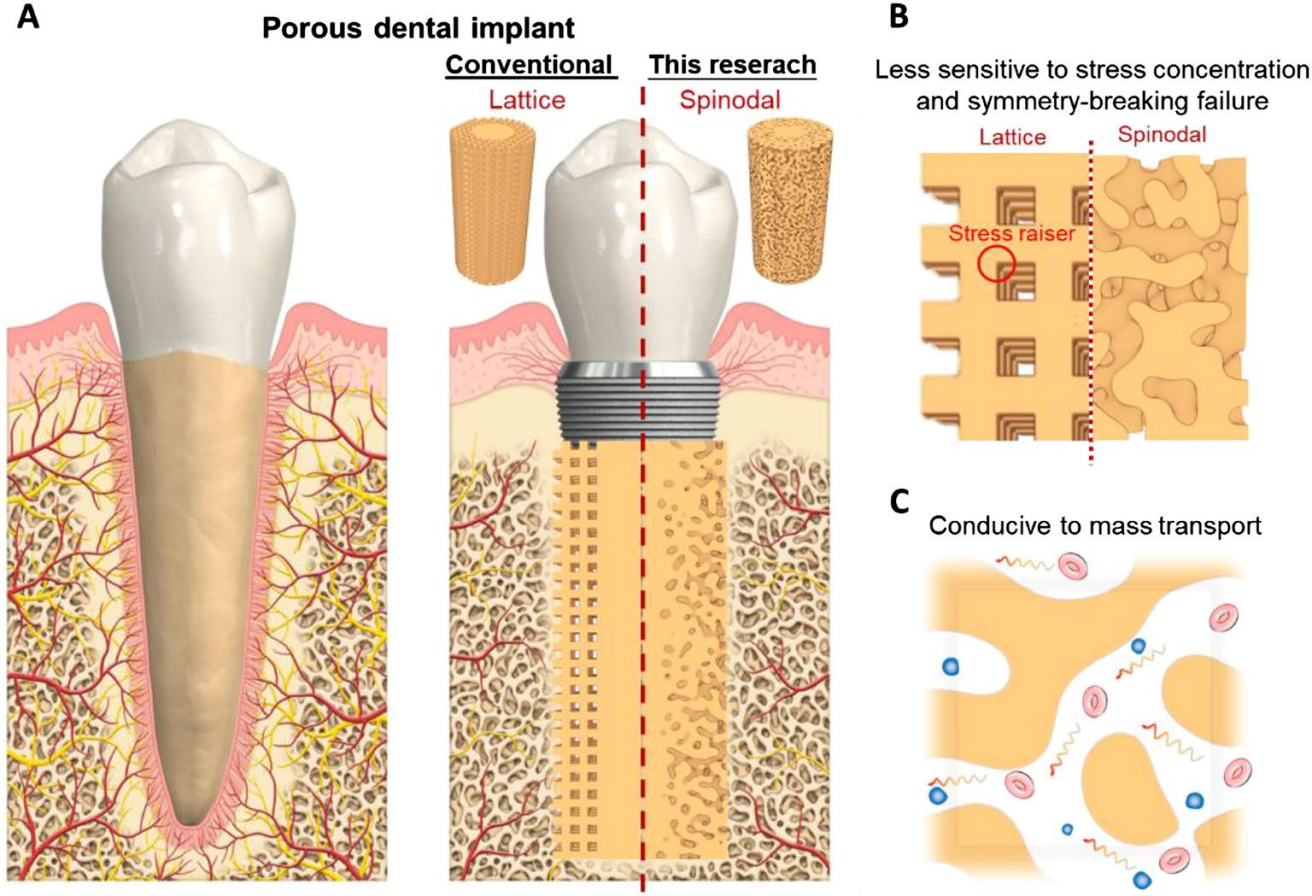
Comparison between lattice and spinodal structure for dental implant application. (**A**) Lattice and spinodal structure based dental implant for replacing natural tooth; (**B**) comparison of mechanical properties; (**C**) good biological property of spinodal structure. By having a stochastic yet interconnected, smooth, and constant pore channel, the spinodal structure is not only morphologically close to natural spongy bone, but also functionally superior from a combined mechanical and biological perspective. Dental implant image adapted from Ref.^22^.

Due to the insufficiencies of application of autograft (patients’ own tissue) and allograft (taken from another person), biomimetic materials and structures play a pivotal role in tissue engineering for effective bone repair and replacement^1^. Porous materials with regular structures, such as rod- or plate-based lattices^2–4^ and triply minimal periodic surface (TMPS) structures^5–7^ have been extensively studied in biomaterials design. This is largely because of their ease of structure parameterization and high controllability. In striking contrast, there were quite limited efforts on designing stochastic biomaterials with tailored structure and property. Among different kinds of stochastic porous materials^8^, spinodal materials^9,10^ is of particular interest due to its intriguing combination of bi-continuity and special stochasticity. The spinodal structure originates from the thermodynamic process of spinodal decomposition, in which a metastable phase self-separates into two distinct phases upon thermal treatment^11^. The resulting bi-phase structure displays an interpenetrating, co-continuous, and stochastic morphology, characterized especially with rather uniform feature size and smooth phase interface (close-to-zero mean curvature^12^) throughout the structure. The spinodal porous materials is obtained by assigning one phase as solid material while the remaining as void. Its special spinodal architecture brings not only distinctive mechanical properties (e.g. high specific strength, insensitivity to imperfection and symmetry-breaking failure common in regular structures), but also favored biological property with good mass transport. The above characteristics make the spinodal materials highly promising for a broad range of applications, such as impact protection system^9,13^, microreaction medium^14^, electrochemical sensor^15^, and, in particular, tissue engineering^16–18^ with both strict mechanical and biological requirements. For instance, the vast majority of orthopedic implants demand not only excellent mechanical function to bear physiological loading^19^, but great biological function to promote nutrient transport, cell proliferation, bone-implant bonding, and thus long-term implantation success^20,21^. The spinodal structure with uniquely combined mechanical and biological properties opens exciting possibilities for fabricating various orthopedic implants; see the dental implant example in Fig. 1. Despite the huge potential of spinodal structure in biomedical application, efficient generation and design of the spinodal biomaterials remains elusive because of the extreme structural complexity in its nature.

There are two main techniques to generate porous structures with guaranteed spinodal morphology. A typical approach to generating spinodal structure is utilizing a physics-based model (e.g. phase field model^23^ and lattice Mote Carlo approach^24^) to simulate spinodal decomposition process, from which one can derive spinodal structures with the expected bi-continuity and randomness. However, physics-based simulation is known for its prohibitively high computational cost by solving complex differential equations. In that sense, some researchers followed the seminal work by Cahn^25^ and used mathematical model to approximately describe the spinodal structure^26,27^; that is, spinodal structure at the initial stage of spinodal decomposition can be approximated by superposition of many random sinusoidal functions. However, the computation time of the mathematical generation model can increase rapidly with the generation size; because a huge number of random sinusoidal functions (typically *N* = 10000^28^) are required for best approximation of the spinodal structure. In addition, due to its random nature and as a simplified approach, the mathematical approximation method has no capability in generating, for example, periodic spinodal structure^26^. Periodic structures are however critically needed in many computational design scenarios, such as representative volume element (RVE) study for computing effective properties^29,30^, discrete Fourier transform based microstructure characterization (e.g. n-point statistics^31^) and mechanical modeling^32,33^ for computational efficiency. Also, direct generation of a gradient structure is impossible, since the mathematical approximation model can only generate a homogeneous structure with a specific porosity fraction at a time. It relies on stitching spinodal structures with different porosities together to yield a gradient one.

To overcome the above shortcomings, this research proposes a data-driven approach for generating spinodal structure, based on a fully convolutional neural network (CNN). Conventionally, CNN is a type of artificial neural network broadly used in computer vision (CV) tasks, such as image classification/labeling^34^, image segmentation (pixel-wise labeling)^35,36^, and object localization^37^. CNN typically has a hierarchy of convolutional layers, which essentially plays a role of non-linear dimension reduction of high-dimensional pixel-based images^38^ (compared to the linear theory-based principal component decomposition^39^). After dimension reduction through distilling salient features of image, CNN facilitates performing downstream CV tasks by avoiding explicitly dealing with raw pixel-based images. With the distinctive feature extraction and image modeling capability, CNN and its variants have been increasingly applied for microstructure evolution simulation in material science and engineering^40,41^. Specifically, microstructures are fed as pixel-based images, and CNN learns the mapping relationship between the original and evolved microstructures. After training, CNN can effectively replace the physics model for quick but realistic simulation of microstructure evolution.

Motivated by recent success on CNN-enabled fast microstructure evolution simulation, we hereby propose to use CNN to substitute the cumbersome physics-based phase-field (PF) model for spinodal decomposition simulation and, therefore, for efficient generation of spinodal structures. It unifies merits of the two existing approaches, i.e. the flexibility of physics-based model and computational efficiency of mathematical approximation model in generating spinodal structures. Specifically, the proposed CNN-based approach has three-fold attributes: (1) flexibility in generating realistic spinodal structures based on dynamic spinodal decomposition simulation instead of approximating spinodal structures. Particularly, circular padding of CNN can effectively mimic the periodic boundary condition in a physics-based simulation, thereby allowing for generation of periodic spinodal structures; (2) computational efficiency in simulating spinodal decomposition and thus generating spinodal structure, by simply treating structure evolution as a structure-to-structure regression problem; (3) spatial scalability to generating spinodal structures with large and variable dimensions, without having to retrain CNN. The following sections present the principles of the newly developed CNN method as well as different case studies, which demonstrate the above-mentioned attributes such as flexibility, efficiency, and scalability of the proposed CNN-based approach in generating and designing various spinodal biomaterials.

## Results

### Training of CNN for substituting phase-field model

**Figure 2:**
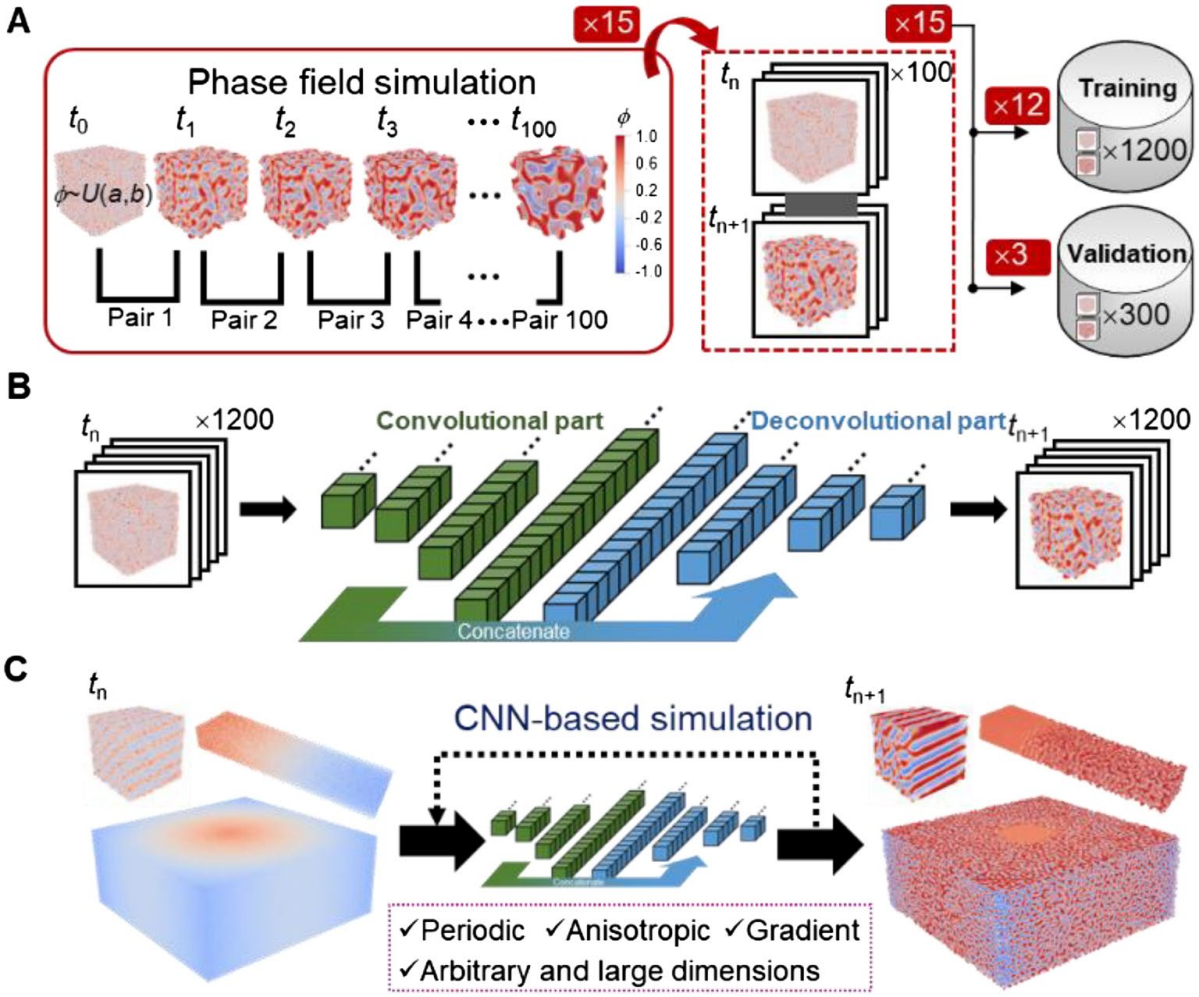
The proposed CNN approach for generating spinodal structures; (**A**) data generation from phase-field based spinodal decomposition simulation; (**B**) CNN training using the generated input–output pair dataset; (**C**) as-trained CNN for generating various structures from CNN-based spinodal decomposition simulation via iterative prediction.

A deep 3D CNN with a fully convolutional architecture is trained to learn the spatio-temporal evolution dynamics of spinodal decomposition and replace the phase field model for fast generation of spinodal structures. To provide corresponding dataset, we first perform a total of 15 phase-field simulations at the resolution of 64 × 64 × 64 voxel^3^. As illustrated in Fig. 2(A), they all start with random initialization

1 $$\left. {{\varvec{\phi}}\left( {\varvec{x}} \right)} \right|_{t = 0} \, \sim \,U\left( {a,b} \right)$$

where *ϕ* is the phase variable describing the phase field, *a* and *b* are respectively the lower and upper bound of the random noise, *U*. The range of the initial noisy phase field is held constant, i.e., $b - a = 0.3$ throughout this study, and the mean of the noise, $\mu = \frac{a + b}{2}$, can vary from -0.7 to 0.7, which permits obtaining spinodal structures with a wide range of porosity fractions. Specifically, the spinodal structure will be extracted from the simulation result by using a threshold value: $\phi_{threshold} = 0$, namely ${\varvec{\phi}}\left( {\varvec{x}} \right) > 0$ for solid phase and ${\varvec{\phi}}\left( {\varvec{x}} \right) < 0$ for pore phase. Therefore, a noisy phase field with large mean $\mu > 0$ (biased towards solid phase) will develop into a dense spinodal structure with less porosity. Note that, in addition to such solid-based spinodal structure, one can also extract shell-based spinodal structure^10^ by assigning phase interface a certain thickness and the whole remaining as voids. For illustration purpose, this research would exclusively focus on solid-based spinodal structure.

To generate a training dataset with maximum variability, we use initialization with equi-spaced mean for the 15 simulations, i.e., *μ* = − 0.7, − 0.6, …, 0.7. Phase field results of neighboring time steps are then extracted as input–output pairing data, see Fig. 2(A). In doing so, the adopted 3D CNN with an convolutional-deconvolutional architecture would basically learn the general evolution relationship between structures at *t*_n_ and *t*_n+1_ as shown in Fig. 2(B). The trained CNN can thus predict next-step structure developed for any structure input. Fig. S1 presents the training and validation curves during training 3D CNN. As training goes on, we will achieve convergence after approximately 30 epochs, and eventually reach a small training and validation error of MSE = 1.45 × 10^−5^ and 3.71 × 10^−5^, respectively. Therefore, the trained CNN is able to accurately predict the spinodal structure evolution. However, it is pointed out that the rather small error is for prediction of one-step evolution, instead of long-term dynamic simulation. The essential purpose of training such a 3D CNN is to well reproduce multi-step dynamic spinodal decomposition given any initial random phase field at *t*_0_, thus deriving realistic spinodal structures. The trained CNN can simulate the dynamic spinodal decomposition process like a phase field model, i.e., taking a random field as input at *t*_0_, and predicting the next-step field in an iterative way till the completion of entire simulation, as indicated by the dashed arrow in Fig. 2(C). Next, testing of the above-trained 3D CNN for simulating long-term, multi-step spinodal decomposition is conducted, and the generated structures with accurate spinodal morphology is quantitatively demonstrated.

### Testing on generating structures with accurate spinodal morphology

**Figure 3:**
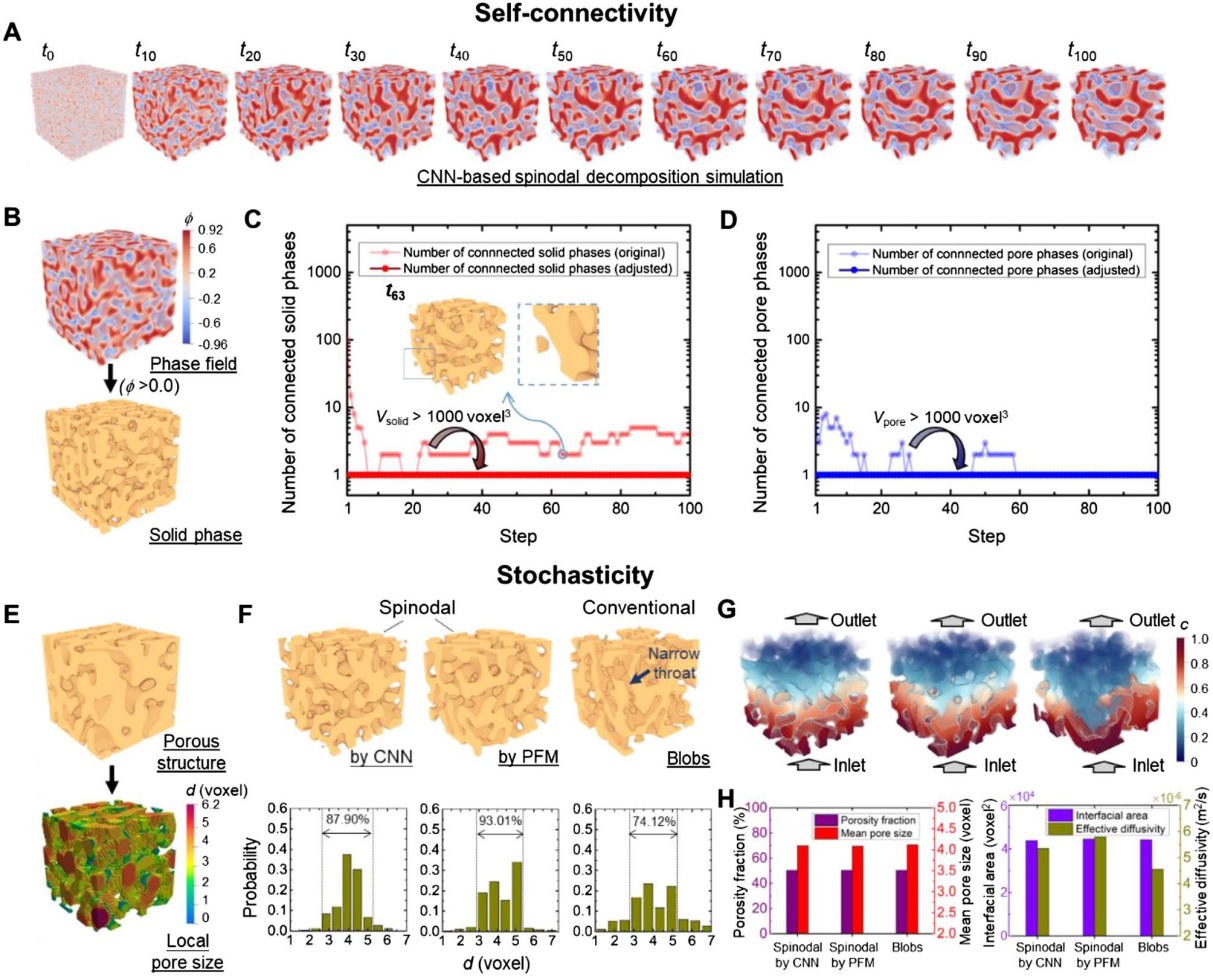
Testing of the trained CNN on accurately generating spinodal structure with special self-connectivity and stochasticity. (**A**) an example of full CNN-based spinodal decomposition simulation; (**B**) extraction of two-phase spinodal structure (i.e., voxelization) from raw simulation result; number of (**C**) connected solid phases and (**D**) connected pore phases within the evolving spinodal structures throughout the spinodal decomposition process; (**E**) illustration of local pore size for a spinodal structure by calculating the local thickness; (**F**) pore size distribution for two spinodal structures generated by CNN and PFM, as well as a conventional stochastic porous structures as benchmark; (**G**) diffusion modeling result of concentration distribution for the three porous structures; (**H**) summary of geometrical characteristics and transport property of the three porous structures.

In this subsection, the trained CNN on accurately reproducing spinodal structures with strict self-connectivity, special stochasticity, and resultant good transport property is examined. Figure 3(A) show an example of full spinodal decomposition simulation via iterative prediction based on the trained CNN. By inspecting opposing boundaries in the modeling results, we can see that periodic boundary condition has been successfully applied through circular padding. Fig. S2 gives a more detailed illustration by using the step-50 result as an example. The characteristic feature size of spinodal structure increases as spinodal decomposition proceeds, but structure evolution and morphology change clearly slow down at the late stage of spinodal decomposition. After completing the spinodal decomposition, one extracts two-phase high-contrast spinodal structures from the raw phase field results as depicted in Fig. 3(B). Note that, a complete simulation would generate a series of spinodal structures, and those structures at the early stage of spinodal decomposition would have a smaller characteristic feature size, e.g., slim ligaments and narrow pore tunnel. We may extract spinodal structure at steps of interest depending on the design objective. To demonstrate the strict bi-connectivity of structures obtained, we simply compute the number of self-connected solid and pore phases in derived structures for the entire spinodal decomposition process; see the light red and blue lines in Fig. 3(C,D). There however clearly exist more than one connected solid or pore phases for the derived spinodal structures, and this is especially true for structures obtained at the beginning of the spinodal decomposition. More than one connected phase exist mainly due to numerical artifacts associated with periodic boundary condition; see inset in Fig. 3(C). Those disjoint particles are “connected” to the structure on the other side of the volume through the periodic boundary, but would exist in isolation physically. Such an event is more common for the early extracted spinodal structures due to their dense ligaments; see spinodal structure for step 1 in Fig. S3. After we filter out the tiny numerical artifacts using a thresholding volume of 1000 voxel^3^, main architecture with strict bi-connectivity in 3D is obtained, as indicated by the only one connected pore and solid phase for every derived structure; see dark red and blue lines in Fig. 3(C,D).

Besides the self-connectivity, another pronounced feature of spinodal structure is its signature stochasticity featuring very smooth and constant pore channel, which would result in outstanding biological properties for tissue engineering applications. Here we quantitatively prove the good transport and biological properties of the structure by CNN-based spinodal decomposition simulation. For reference purposes, we provide spinodal structure by PF modeling as standard one and another typical stochastic structure, blobs described by Gaussian random field (GRF)^42^, to highlight the excellent transport properties of spinodal structures. The local pore size of porous network is first calculated for the three structures; see an illustration of local pore size in Fig. 3(E). Pore size distribution is summarized and compared in Fig. 3(F). Note that, for fair comparison, three structures are strictly controlled with the same overall porosity fraction of 50% and mean pore size of 4.1 voxels. We can see that spinodal structure by CNN well replicates the rather constant pore channel of the one by PF modeling. Both spinodal structures shows pore size mainly lying in between 3 and 5 voxels while almost free from very small throats; i.e. *d* < 1.5 voxels. However, the blobs structure displays more variable pore channels, as characterized by less concentrated distribution of pore size in Fig. 3(F). One can also easily find the narrow channel (indicated by the arrow) and great variation of channel size on the surface of the blobs structure, but both spinodal structures show smooth and less varied pore channel beneficial to bio-transport. To further investigate their transport property, diffusion modeling is performed (Fig. 3G), since the process of nutrient transport and cell migration into the porous matrix can be considered as a diffusive process^43^. The two spinodal structures have an effective diffusivity of 5.35 × 10^−6^ m^2^/s and 5.79 × 10^−6^ m^2^/s respectively, which are clearly higher than 4.56 × 10^−6^ m^2^/s of the blobs structure. In summary, the spinodal structure by CNN well reproduces the spinodal stochasticity of the PF modeling derived spinodal structure, and thus exhibits stronger diffusion transport than the conventional stochastic materials at the same porosity fraction, mean pore size, and resulting interfacial area; see Fig. 3(H).

### Testing on spatial extrapolation for generating large spinodal structure

The capability of generating large spinodal structures of arbitrary dimensions is important for designing orthopedic implants with a variety of sizes. Although technically the fully convolutional architecture of the adopted CNN allows for taking input of variable sizes, its spatial scalability to correctly modeling large-scale spinodal decomposition is unknown or unsubstantiated. In this section, the spatial extrapolation of the as-trained CNN to modeling spinodal decomposition and thus deriving spinodal structure of large dimensions is demonstrated. By using the earlier trained CNN, three groups of spinodal decomposition simulations are performed at the original modeling size of 64 × 64 × 64 (benchmark as standard spinodal structure) and two large modeling sizes of 96 × 96 × 96 and 128 × 128 × 128.

**Figure 4:**
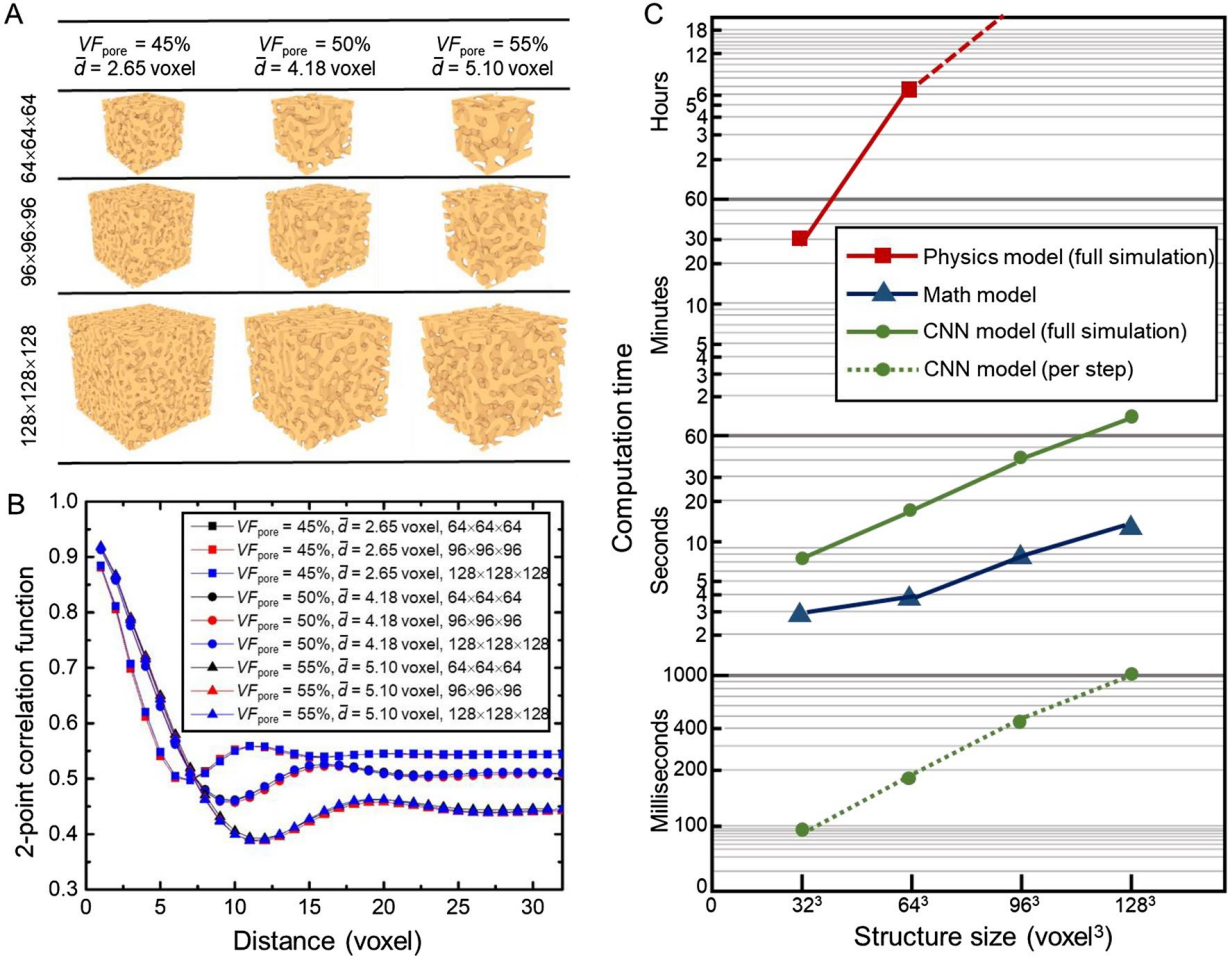
Spatial extrapolation of the as-trained CNN to generating large spinodal structures. (**A**) spinodal structures of three sizes are generated for different porosity fractions and mean pore size; (**B**) 2-point correlation statistics of the generated spinodal structures; (**C**) A comparison of computation time of different models in generating spinodal structures of different size. Note that both physics model and CNN model generate spinodal structure through dynamic spinodal decomposition simulation. A full simulation with 100 steps is carried out for both of them. The computation time is measured in the same computational environment for the three models: 36-core Intel Xeon Gold 6154 CPU, 2 × NVIDIA Tesla V100 GPU, 180 GB RAM.

Figure 4(A) presents the correspondingly derived spinodal structures. For quantitative comparison, we then calculate 2-point statistics for those structures. Compared with some simple hand-designed descriptors, e.g., mean grain size for a grain structure and porosity fraction for a porous structure, 2-point statistics provide a higher-order and more complete statistical characterization of stochastic structures^31^. Figure 4(B) shows that 2-point statistics curves overlap tightly with each other for spinodal structures of different sizes. CNN consistently generates large structures that are completely statistically equivalent to the spinodal structure of 64 × 64 × 64 dimensions for different volume fractions and mean pore sizes. Therefore, the as-trained CNN can accurately generate standard spinodal structure of large sizes when directly applied to modeling large-scale spinodal decomposition simulation. It is hypothesized that the spatial scalability of the as-trained CNN essentially lies in its accurate learning of evolving kinetics of spinodal decomposition, which is independent from modeling size and is uniform over the modeling domain.

To demonstrate the computational efficiency of CNN, we further test the computation time of the physics model, mathematical model, and CNN model in generating spinodal structure of different sizes; see Fig. 4(C). Physics model is a finite element (FE) based phase-field model executed in the commercial COMSOL Multiphysics package, the mathematical model is based on the recent approximation model^27^ for generating anisotropic spinodal structures implemented in GIBBON MatLab toolbox^44^, and the CNN model is the proposed approach in this research. It can be seen that the CNN model generally achieves ~ 3 orders of magnitude speed-up than the physics model. Its structure generation efficiency is also comparable to that of the mathematical approximation model, considering that a 100-step full simulation would actually give a stack of spinodal structures. For the four tested modeling sizes, CNN model takes only a fraction of a second per step. In brief, our CNN model provides an alternative way to generate realistic spinodal structures like a physics-based approach, but at a generation speed comparable to a mathematical approximation model.

### Application 1: design spinodal bone structure with target anisotropic elasticity

**Figure 5:**
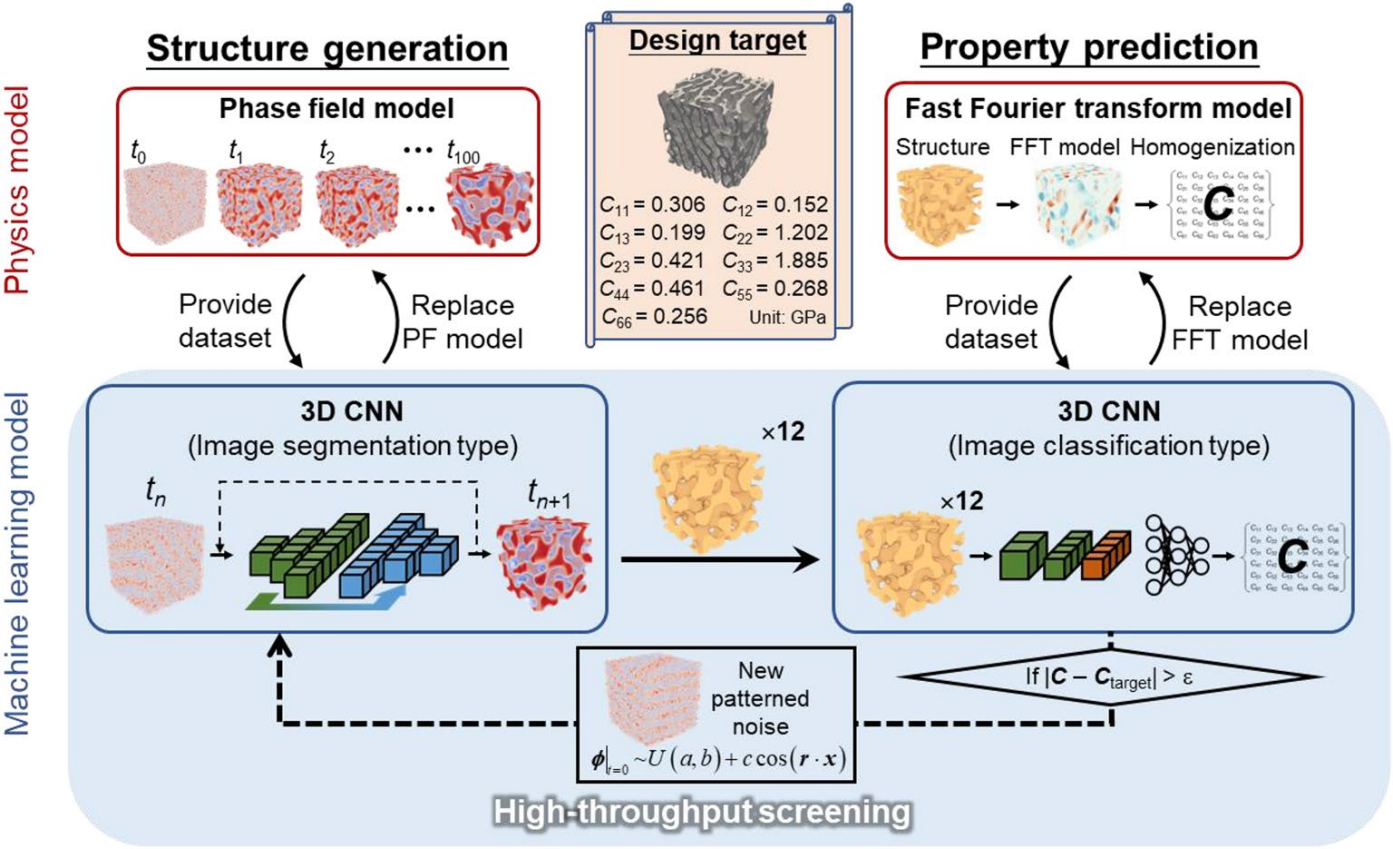
CNN-based high-throughput screening for spinodal structure with target anisotropic elasticity. Besides the CNN used to replace phase field model for quick spinodal structure generation (left-side), an image-classification type CNN is trained to substitute FFT-based mechanics model for ultrafast property prediction (right-side). The two CNNs work in a fully integrated way, enabling high-throughput generation and screening of large amount of spinodal structures.

This section gives a detailed application of the above trained CNN for designing spinodal bone structure. This application shows its importance of efficiently generating periodic and anisotropic structures. One of the main requirements for a medical implant is to match the mechanical property of the host bone, which varies with the anatomical site, patient age and bone condition. On-demand design given any target bone property is thus required. As an example, we design artificial bones based on anisotropic spinodal structures. The aim is to closely match the anisotropic elasticity of the natural bones measured in Ref.^4^, as described by the elastic stiffness tensor ***C***; see design target in Fig. 5. A problem now arises, since one can only obtain spinodal structures with or near isotropic properties by using the uniform noise initialization based on Eq. (1). To introduce anisotropy in a simple manner, we use strongly patterned noise as the initial phase field for spinodal decomposition simulation

2 $$\left. {\varvec{\phi}} \right|_{t = 0} \, \sim \,U\left( {a,b} \right) + c\cos \left( {{\varvec{r}} \cdot {\varvec{x}}} \right)$$

where the initial phase field is now basically a summation of a completely random noise and a 3D cosine plane wave. ***x*** denotes the cartesian coordinates in 3D space. The direction of the plane wave and thus anisotropy of developed spinodal structures are controlled by directionality vector, ${\varvec{r}} = \left( {r_{1} ,r_{2} ,r_{3} } \right),\left| {\varvec{r}} \right| = 1$. $c \in \left( {0,0.015} \right)$ further decides the strength of applied pattern and hence anisotropy level of spinodal structures. Figure 4 depicts the overall computational design framework for high-throughput screening of a desired spinodal structure. It is composed of the proposed CNN for generating anisotropic spinodal structures using the above Eq. (2) and another CNN for predicting the corresponding properties. To train the second CNN, massive periodic spinodal structures are first generated by our structure generation CNN and serve as input to an FFT-based mechanical model to generate the structure–property pairing dataset; see Fig. S4 for the detailed dataset generation process. Eventually, by replacing the physics models, the two efficient 3D CNNs work together to enable high-throughput screening, i.e., quick generation of different spinodal structures and examination of their properties until finding out the one with |***C***-***C***_target_|< *ε* upon further verification by FFT-based mechanical model.

**Figure 6:**
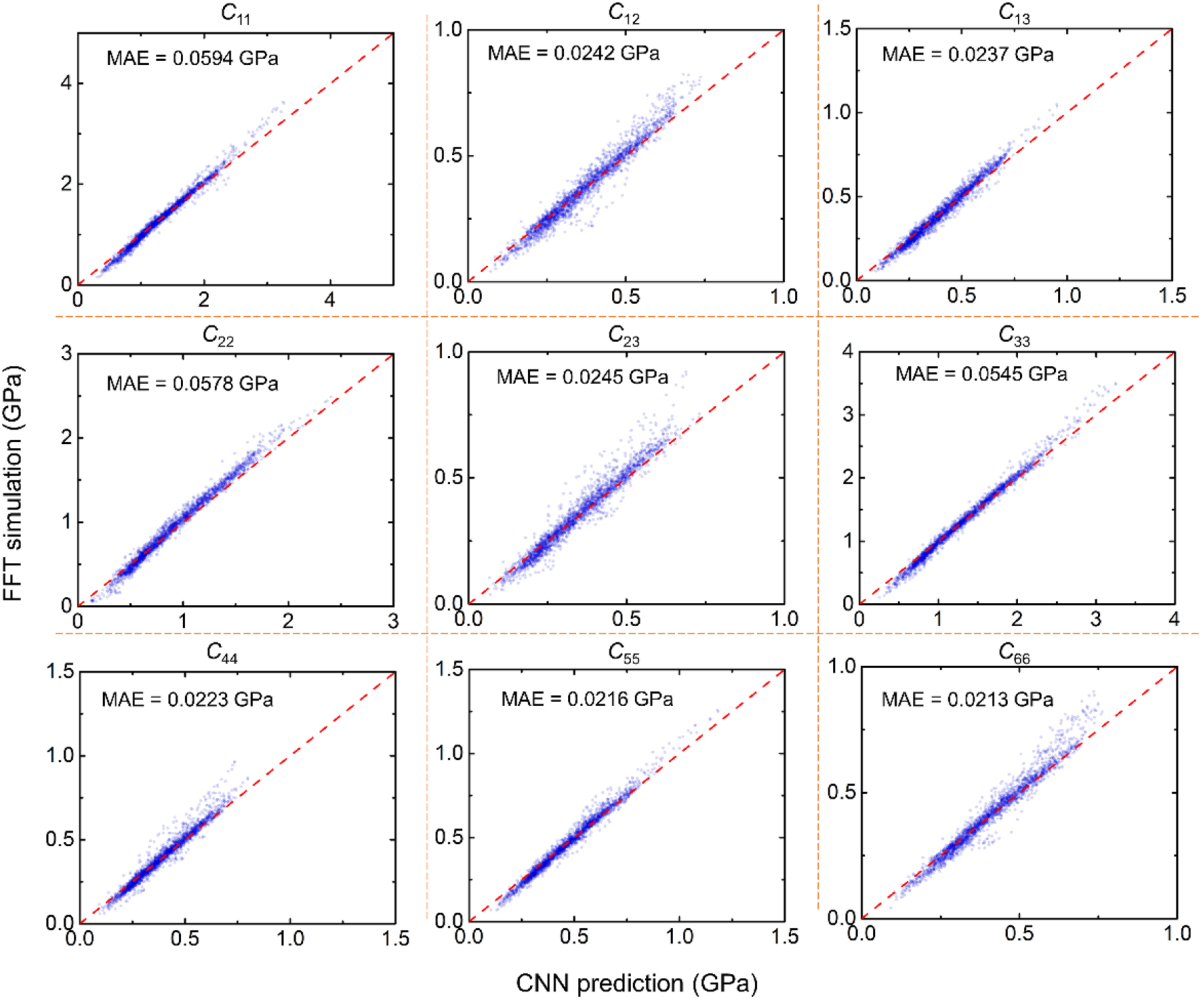
Testing results of the trained 3D CNN for predicting elastic stiffness tensor, ***C***. The respective MAE for predicting each of the nine stiffness components is also indicated.

Figure 6 shows the prediction of stiffness tensor, ***C***, using the trained CNN against FFT-based mechanics simulation results. By focusing on major elastic moduli components, we consider the studied structure as orthotropic. A small overall mean absolute error (MAE) of 0.0344 GPa in predicting ***C*** is achieved. In each subfigure, we also indicate the detailed MAEs in predicting each stiffness component, which are 0.0594, 0.0242, 0.0237, 0.0578, 0.0245, 0.0545, 0.0223, 0.0216, and 0.0213 GPa for *C*_11_, *C*_12_, *C*_13_, *C*_22_, *C*_23_, *C*_33_, *C*_44_, *C*_55_, and *C*_66_, respectively. We note that there tends to be some outliers with relatively large predictive errors in the regime of high stiffness. In part, this is attributed to the imbalanced dataset resulting from the random initialization method by using Eq. (2). Fig. S5 presented the distribution of data points for each *C*_*ij*_ dataset. The data points are concentrated in the low-to-medium stiffness regime, clearly showing skewed distributions instead of uniform ones in an ideal case. Therefore, the current initialization method favors generation of highly porous spinodal structures with relatively low stiffness.

**Figure 7:**
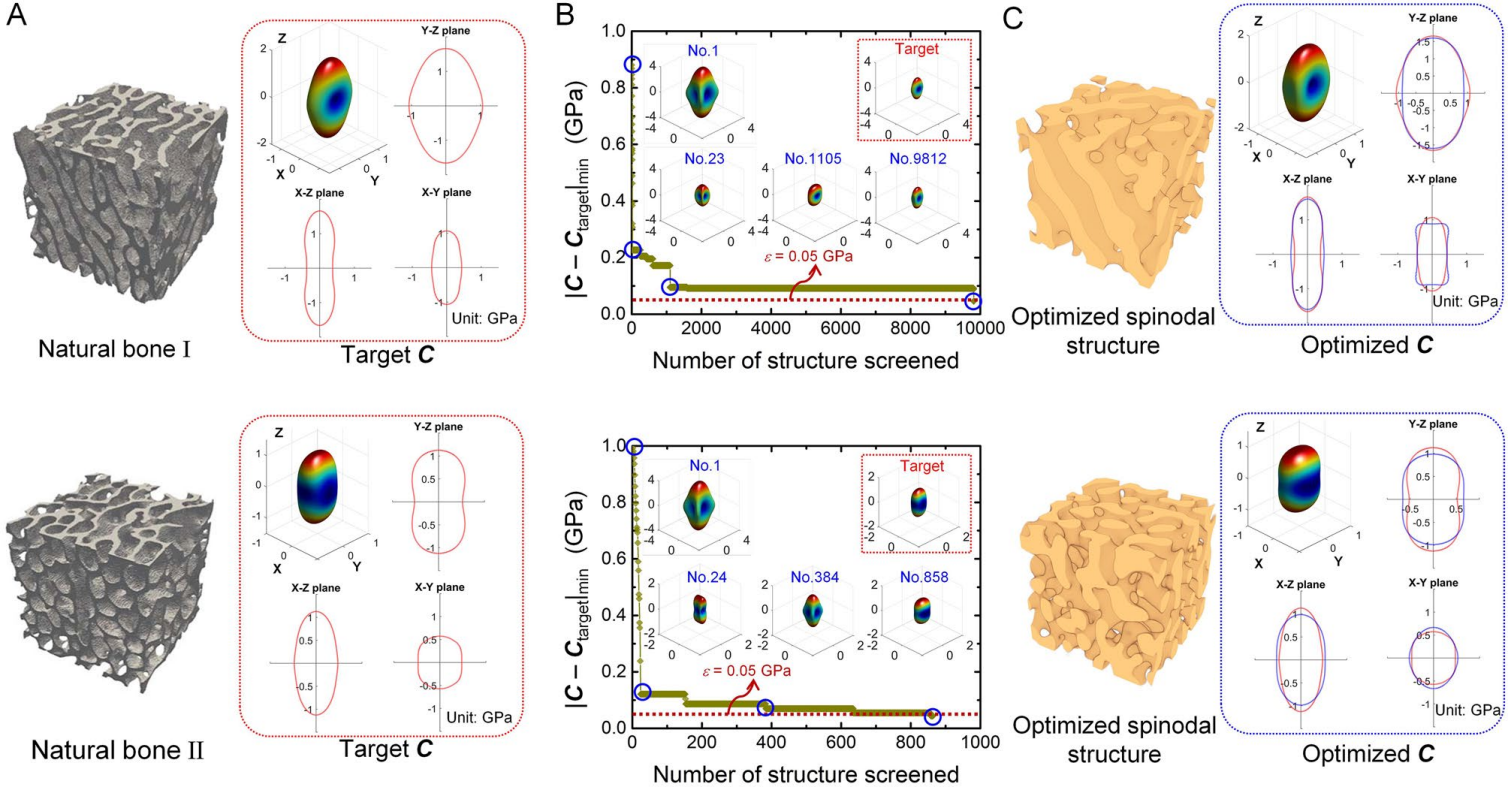
(**A**) natural bone (design target) and its anisotropic elasticity represented by 3D elastic surface and its 2D projections; (**B**) high-throughput screening process; (**C**) high-throughput screening results, namely the optimized spinodal structures and their anisotropic elasticity (blue lines) compared to that of natural bones (red lines).

To design spinodal structures with a close mechanical match to natural bone, a small allowable difference of *ε* = 0.05 GPa is used in the design function. Figure 7(A) shows the two natural bones and their anisotropic elasticity as the design targets. Figure 7(B) depicts the high-throughput screening process, which takes a total of 26 min for searching the two desired structures. Since we use random screening, rather different number of spinodal structures have been screened till finding out the two optimal spinodal structures. Figure 7(C) compares designed spinodal structures and natural bones in terms of 3D directional Young’s modulus. According to Wolff’s law^45^, bone resorbing and remodeling would occur in response to external stimuli, e.g., biomechanical loading. The natural bone structure thus sometimes shows clear orientation possibly along the stress trajectory, such as bone I herein. Similarly, the corresponding spinodal structure displays oriented morphology by reproducing the mechanical property of the nature bone. We point out that the feature size of the optimized spinodal structure is larger than that of the natural bone, suggesting that feature size can be further considered during optimization to design spinodal structure with maximum morphological match. From the mechanical point of view, the current spinodal structures achieve great mechanical resemblance to the natural bone, as indicated by 3D direction-dependent Young’s modulus. The visualized 3D elastic surface and its two-dimensional projections along three coordinates axis shows a good agreement between the natural bone structure and the designed spinodal structure. Therefore, the CNN-based high-throughput screening framework can serve to design any target anisotropic spinodal bone structures on demand. It demonstrates the strength of the synergy between contemporary machine learning models in achieving structure design, without the need for structure parameterization that is a long-standing, common roadblock in designing various stochastic materials.

### Application 2: design large spinodal structure with desired gradient porosity

**Figure 8:**
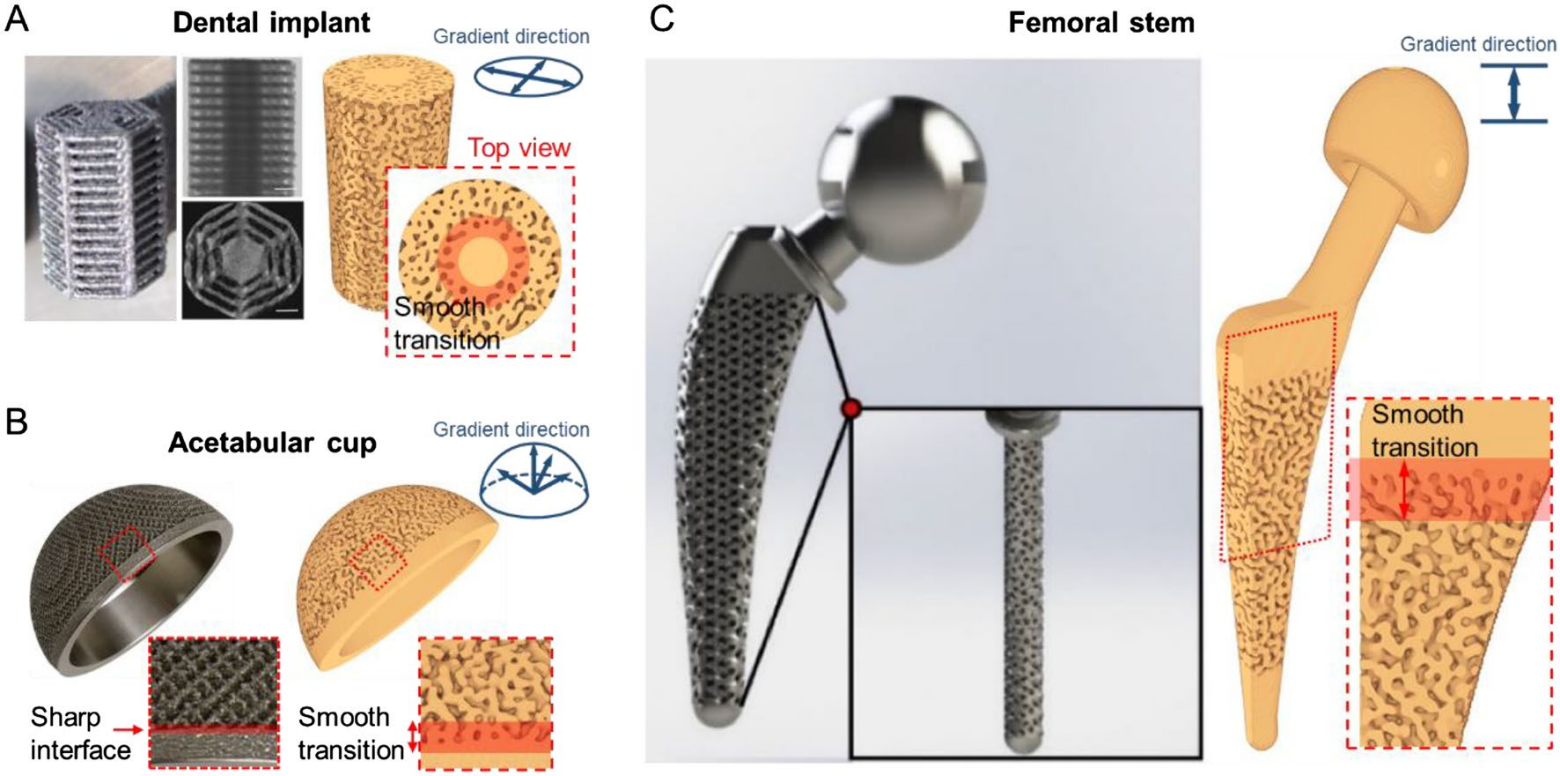
CNN-enabled generation of large gradient spinodal structures for fabricating various porous orthopedic implants: (**A**) dental implant; (**B**) acetabular cup and (**C**) femoral stem of hip implant. Images of the real implants are adapted from^46–48^.

This subsection further shows the practical significance of the trained CNN in directly generating large and gradient spinodal structures. Large porous structures with gradient porosity are a common practice for various types of orthopedic implants. For example, an ideal dental implant usually requires solid core to bear occlusal loading, but highly porous structure on the surface to minimize implant-bone stiffness mismatch and encourage bone ingrowth; see Fig. 8(A). This is the same for the acetabular cup of a hip implant, where surface porosity can improve bone-implant bonding while the overall structure requires sufficient mechanical strength; see Fig. 8(B). As for the femoral stem of a hip implant, the distal tip is inserted into the medullary cavity and the main part interacts with trabecular/cancellous bone. Therefore, porous structure is required in relevant regions to reduce stress shielding; see Fig. 8(C). To produce spinodal analogues of those gradient porous structures, we perform large spinodal decomposition simulations starting with correspondingly gradient random fields (see Fig. S6), followed by cutting as-obtained gradient structures into required shapes. The proposed design workflow for designing spinodal implants with both desired external shape and internal structure is depicted in Fig. S7. As shown in Fig. 8, next to the real orthopedic implants are the finally obtained spinodal replicates. It can be found that the distribution of porosity can be precisely controlled to design spinodal structure with different types of gradient porosity. More importantly, unlike the lattice-based implants with sharp porosity change at the interface, seamless transition is satisfied between porous and fully solid regions; see red areas highlighted in Fig. 8(A−C). The width and smoothness of transition region can be further adjusted by accordingly changing the initial random field for CNN-based simulation; see Fig. S8. When compared with the mathematical approximation model, CNN directly generates complex gradient structures and avoids much human intervention during gradient structure generation. Nonetheless, the mathematical approximation model can only generate homogeneous structures with a predefined porosity level. It requires laborious post-processing to join spinodal structures with different porosities to form a gradient one. The CNN approach thus provides a straightforward means to generate high-quality large-piece spinodal-based gradient biomaterials.

## Discussion

We pointed out that, for illustration purpose, a rather simple way to generate and design anisotropic spinodal structure is adopted based on Eq. (2). By doing so, the anisotropy of developed spinodal structure just inherits from the non-homogeneous random initialization of two phases, and the spinodal structure essentially evolves in an isotropic manner during the entire spinodal decomposition. A more rigorous method to generate anisotropic spinodal structure is to enforce direction-dependent microstructure evolution kinetics, by which anisotropic spinodal structure forms more naturally from directional spinodal decomposition. This is done by using anisotropic surface energy in a physics-based phase field model^49^. To develop a corresponding data-driven substitute, one will have to train a multi-input CNN^40,50,51^ that takes anisotropic surface energy as additional inputs, since the evolved structure is now conditioned on both the original structure and the surface energy. Consequently, phase-field based spinodal decomposition simulation for different anisotropic surface energies should be performed to provide proper dataset, which allows CNN to correctly learn the relationship between microstructure evolution and anisotropic surface energy.

In terms of multi-step temporal prediction, the accumulating error of CNN prediction against ground truth/physics simulation is a known issue^40^. The problem can become even more serious when we predict into far future, i.e. extrapolative prediction along the time axis. With the increasing success of physics-informed machine learning (PIML) in various fields^52^, physics-informed neural network is anticipated to alleviate or even solve the forgoing issue, by improving the generalization and robustness of the trained CNN while using little to no training data.

The CNN-based approach can be further integrated with other topology optimization algorithms, enabling design of large spinodal structure with locally fine-tailored morphology. For example, Senhora et al.^53^ uses four spinodal structures generated by the mathematical approximation method as elementary structures, and design large spinodal structure by optimizing local existence, orientation, and porosity of the four candidate spinodal structures. Li et al.^26^, instead of using only four basis spinodal structures, optimized parameters of the mathematical approximation spinodal model of each local spinodal structure. Therefore, the candidate spinodal structure basically covers the entire structure space by the mathematical approximation method. The above facts imply the great potential of the proposed CNN on integration with other topology optimization methods in a similar way. Furthermore, by replicating the tremendous structure generation flexibility of a physics-based model, CNN can provide a richer space of basis spinodal structures than mathematical approximation model.

## Conclusion

In conclusion, while CNN-enabled ultra-fast microstructure evolution simulation has achieved much success recently, in this research, it is newly proposed for the spinodal decomposition process and, for the first time, towards solving a practically significant problem: generation and design of high-performance stochastic biomaterials. The presented CNN model with circular padding can accurately simulate the spinodal decomposition and generate various spinodal structures, as does a physics-based phase field model. It however takes only seconds to complete one simulation (compared to hours of a physics model in general), and therefore allows for ultra-fast generation of spinodal structures. The 2-point statistics analysis demonstrates that the as-trained CNN is scalable to simulating spinodal decomposition and deriving spinodal structures of arbitrary large dimension, without the need for retraining CNN. The trained CNN, in conjunction with another property prediction CNN, is used to perform on-demand structure design given experimentally measured bone property. The designed spinodal structures accurately reproduce the anisotropic mechanical properties of natural bones. We further present a workflow for designing large orthopedic implants with a desired outer shape and internal porous structure. Three practical implants with different gradient porosities, including dental implant, acetabular cup, and femoral stem, have been successfully generated.

## Methods

### Spinodal structure dataset by phase-field simulation

To provide the relevant training dataset, we perform 15 phase-field simulations in total, with each being 100 step long and at the resolution of 64 × 64 × 64 voxel^3^. In a phase-field spinodal decomposition simulation, phase variable, *ϕ*, is used to describe the phase field of two phases. The phases are considered pure when $\phi = \pm 1$. The phase separation from a random mixture, or essentially spatio-temporal evolution of *ϕ*, is governed by the known Cahn–Hilliard equation

3 $$\frac{\partial \phi }{{\partial t}} = \nabla \cdot \frac{\gamma \lambda }{{\varepsilon^{2} }}\nabla \Psi$$

where *γ* = 1 m^3^⋅s⋅kg^−1^ is the mobility, *λ* = 0.085 N is the mixing energy density, *ε* = 0.08 m is a capillary width controlling the thickness of the interface, and ψ is the total free energy described by

4 $$\Psi = - \nabla \cdot \varepsilon^{2} \nabla \phi + \left( {\phi^{2} - 1} \right)\phi$$

where the first term is the gradient energy concerned with phase interface and the second term describes the chemical free energy. Basically, the spinodal decomposition proceeds by minimizing the total free energy of the bi-phase system.

All simulations start with a completely random phase field as described early by Eq. (1). After phase-field simulation, phase field results of adjacent steps are extracted as input–output pair data, which indicates 100 pairs obtained for each PF simulation; see Fig. 2(A). The whole 1500 pairs are split into training (1200) and validation (300) datasets. Testing of the trained CNN will be performed in terms of its capability on deriving accurate spinodal structure through long-term spinodal decomposition simulation, as detailed in Section “Testing on generating structures with accurate spinodal morphology”.

### Architecture and training of CNN for substituting phase-field model

As shown in Fig. 2(B), the 3D CNN architecture is essentially adapted from UNet^54^, which is originally designed for semantic segmentation of medical images. This is because UNet is not only known as a well-tested image-to-image regression tool with a neat encoder-decoder structure, but as a fully convolutional CNN throughout its architecture. The former makes UNet suitable for the current structure-to-structure regression task upon its extension to 3D, while the latter is critical for developing the spatial scalability by permitting input of variable size. We extend it to 3D by adopting 3D convolutional layer. To enable the generation of periodic structure as RVE, we further imposed 3D circular padding^55^ to enforce periodic boundary. The circular padding is mathematically equivalent to tiling the input first, followed by properly cropping to obtain the input after padding; see Fig. S9. In doing so, the convolutional filter (the green box as shown), when sliding at the boundary, would perform computation based strictly on periodic boundary condition, as does a physics-based model. More details about the implemented 3D CNN architecture, e.g., number and size of convolutional layers, are listed in Table S1.

The loss function is mean squared error (MSE):

5 $$Loss_{MSE} = \frac{1}{N}\sum\nolimits_{i = 1}^{N} {\left| {{\varvec{\phi}} - \overline{\user2{\phi }}} \right|}^{2}$$

where *N* is the number of predictions, ${\varvec{\phi}}$ is the predicted phase field or basically spinodal structure by CNN, and $\overline{\user2{\phi }}$ is the ground truth spinodal structure by phase-field simulation. The CNN is trained for 100 epochs with a batch size of 16.

### Calculation of connected solid and pore phase

The number of self-connected solid and pore phase for derived spinodal structure are calculated using *bwconncomp* function in MatLab R2018b. The *bwconncomp* function is originally designed for finding and counting connected components in binary image. Therefore, it can be easily extended to calculating self-connected solid or pore phase by appropriately representing two-phase spinodal structure in required array format. There are multiple options for 3D connectivity for the *bwconncomp* function. In this research, we strictly define that cubic voxels are connected if their faces touch, while two adjoining voxels having only edges and/or corners touched will not be considered connected.

### Calculation of local pore size

Compared to the discrete/isolated pores, it is quite subjective to define pore size for open-cell structures, like the spinodal structure in the current study. Here we select to calculate the pore size for a pore voxel as the local thickness. The local thickness at a point is defined by drawing a sphere containing the point. Then its local thickness is equal to the radius of the largest circle possible in the pore channel; see 2D illustration in Fig. S10. To calculate the local thickness, we use *local_thickness* filter in the quantitative pore analysis toolset – Porespy^56^.

### Diffusion modeling

Pore network modeling (PNM) is used to simulate diffusion in porous materials in this research. Instead of resolving the detailed morphology, PNM treat the porous matrix as a simplified network of pipes. As an example, Fig. S11 compares FEM meshing and the pore network representation of 2D porous structure. PNM can simulate diffusion and other transport process in porous media with computational ease, yet still respecting the pore-scale structure.

In this study, we choose to model the typical gas flow to examine the general diffusion property of our porous structures. As shown in Figs. S11B and S12, once we have constructed the pore network for the porous materials, the molar flow of gas between two adjacent pores connected through the throat/pipe can be represented as

6 $$j_{1,2} = D_{1,2} \left( {c_{1} - c_{2} } \right)$$

where *D*_1,2_ is the effective diffusivity between the two pore 1 and 2, which can be calculated from pore sizes, throat size, and open-space diffusivity^57^, and *c* represents the gas concentration of pore. Equation (6) for all neighboring pores is then assembled yielding a linear system for the whole pore network, by solving which concentration distribution is obtained. The effective diffusivity for the bulk porous structure is finally calculated

7 $$D_{eff} = \frac{jL}{{\left( {c_{in} - c_{out} } \right)CA}}$$

where *j* is the total molar flow through the porous structure, which can be calculated from the modeling result, *L* is the length of structure along the flow direction, *c*_in_ and *c*_out_ are the applied gas concentration conditions at inlet and outlet boundaries, *C* is the molecular density of the gas, and *A* is the cross-sectional area of the porous structure.

### Elastic property dataset by fast-Fourier-transform based mechanics simulation

In spinodal bone design, besides the proposed CNN for quick generation of spinodal structure, an auxiliary 3D CNN (image classification type) is trained to replace fast-Fourier-transform (FFT) based mechanics model for ultra-fast property prediction. To provide structure–property pair dataset with sufficient variability, we start the spinodal decomposition simulation with differently patterned noise and thus generate spinodal structures with varied anisotropy. Each spinodal decomposition simulation is initialized using Eq. (2) with randomly sampled noise mean, *μ*, pattern direction, ***r***, and pattern strength, *c*, as shown in Fig. S13. We utilize the early trained CNN to effortlessly perform 1800 spinodal simulations in total. Note that, since an entire spinodal decomposition simulation, 100-step long herein would give a stack of evolving spinodal structures. Upon the full development of spinodal structure from random initialization, we extract only 12 useful spinodal structures at increasingly spaced time steps, i.e. *t* = *t*_9_, *t*_11_, *t*_14_, *t*_18_, *t*_23_, *t*_29_, *t*_36_, *t*_44_, *t*_53_, *t*_63_, *t*_74_, *t*_86_; because spinodal structures evolves more slowly at the later stage of spinodal decomposition. The FFT-based mechanics model is then utilized to compute corresponding ***C***, and generate structure-***C*** pairs for training and testing use. Compared to the finite-element method, FFT can achieve orders of magnitude speed-up in solving equilibrium equations (mechanical equilibrium equations herein) in periodic systems. This is accomplished by calculating local (mechanical) response of a periodic medium as a convolution integral between the Green function of a linear reference homogeneous medium and a polarization field, proportional to the actual heterogeneity of the fields^33^. Such type of integrals reduces to a simple product in Fourier space.

We implement the FFT-based mechanics model with in-house Fortran code, and eventually get a total of 21,600 structure-***C*** pairs. The whole dataset is split into training (16,800), validation (2400), and testing (2400) datasets as shown in Fig. S13.

### Architecture and training of CNN for substituting FFT-based mechanics model

This CNN would basically build 3D structure-***C*** linkage from the training dataset. The trained CNN can quickly predict ***C*** for any given spinodal structure. Therefore, we adopted a classical image-labeling type CNN to build the relationship between the structure (3D image essentially) and ***C*** (a mechanical “label” of structure). Like most image-labeling CNNs, the adopted CNN herein is made up of convolutional layers for feature extraction and fully connected layers for regression modeling; see Fig. S14. The convolutional layers take images as input and convert it to highly abstract feature maps, and fully connected layers link the features to property, ***C***. It’s known that such a CNN in image classification task usually flatten the features to a long vector at the end of convolutional layers. However, the flattening operation would introduce large fully connected layers that is parameter-intensive. To develop a CNN able to efficiently predict mechanical property, global average pooling is instead appended to the end of convolutional layers. By averaging the feature information, CNN would also leverage the overall structural features of spinodal structure and thus may avoid using noise information for property prediction. The loss function adopted is mean absolute error (MAE):

8 $$Loss_{MAE} = \frac{1}{N}\sum\nolimits_{i = 1}^{N} {\left| {{\varvec{C}}\left( {\phi_{bw} } \right) - \overline{\user2{C}}} \right|}$$

where *N* is the number of prediction, ***ϕ***_*bw*_ is the binary spinodal structure input, ***C*** is the predicted property by CNN, and $\overline{\user2{C}}$ is the ground truth property by FFT mechanics simulation. CNN is trained for 300 epochs, with a batch size of 16.

### Calculation of 3D direction-dependent Young’s modulus

It is difficult to comprehend the variations in elastic property by simply inspecting the 4th-rank or even reduced-order stiffness tensor. Therefore, graphical visualization has been developed and widely used for full appreciation of the properties including the mechanical anisotropy. The direction-dependent effective Young’s modulus is calculated using Eqs. (4, 5)^58^,

9 $$C^{\prime}_{ijkl} \left( {\varvec{d}} \right) = d_{im} d_{jn} d_{ko} d_{ip} C_{mnop}$$

10 $$E^{\prime}_{1} \left( {\varvec{d}} \right) = C^{\prime}_{1111} \left( {\varvec{d}} \right)$$

where is the transformed stiffness matrix along a direction of vector ***d*** (see Fig. S15), is the elastic stiffness matrix, and $E^{\prime}_{1} \left( {\varvec{d}} \right)$ is the directional Young’s modulus.

## Supplementary Information

Supplementary Information.

## Data Availability

The data that support the findings of this study are available from the corresponding author upon reasonable request.

## References

[1] Wang X, Xu S, Zhou S, Xu W, Leary M (2016). Topological design and additive manufacturing of porous metals for bone scaffolds and orthopaedic implants: A review. Biomaterials.

[2] Bastek J-H, Kumar S, Telgen B, Glaesener RN, Kochmann DM (2022). Inverting the structure–property map of truss metamaterials by deep learning. Proc. Natl. Acad. Sci..

[3] Lumpe TS, Stankovic T (2021). Exploring the property space of periodic cellular structures based on crystal networks. Proc. Natl. Acad. Sci..

[4] Colabella L, Cisilino AP, Häiat G, Kowalczyk P (2017). Mimetization of the elastic properties of cancellous bone via a parameterized cellular material. Biomech. Model. Mechanobiol..

[5] Al-Ketan O, Rezgui R, Rowshan R, Du H, Fang NX (2018). Microarchitected stretching-dominated mechanical metamaterials with minimal surface topologies. Adv. Eng. Mater..

[6] Song X, He L, Yang W, Wang Z, Chen Z (2019). Additive manufacturing of bi-continuous piezocomposites with triply periodic phase interfaces for combined flexibility and piezoelectricity. J. Manuf. Sci. Eng..

[7] Zhang M, Yang Y, Xu M, Chen J, Wang D (2021). Mechanical properties of multi-materials porous structures based on triply periodic minimal surface fabricated by additive manufacturing. Rapid Prototyp. J..

[8] Bargmann S, Klusemann B, Markmann J, Schnabel JE, Schneider K (2018). Generation of 3D representative volume elements for heterogeneous materials: A review. Prog. Mater Sci..

[9] Guell Izard A, Bauer J, Crook C, Turlo V, Valdevit L (2019). Ultrahigh energy absorption multifunctional spinodal nanoarchitectures. Small.

[10] Hsieh M-T, Endo B, Zhang Y, Bauer J, Valdevit L (2019). The mechanical response of cellular materials with spinodal topologies. J. Mech. Phys. Solids.

[11] Cahn JW (1965). Phase separation by spinodal decomposition in isotropic systems. J. Chem. Phys..

[12] Jinnai H, Koga T, Nishikawa Y, Hashimoto T, Hyde ST (1997). Curvature determination of spinodal interface in a condensed matter system. Phys. Rev. Lett..

[13] Zhang Y, Hsieh M-T, Valdevit L (2021). Mechanical performance of 3D printed interpenetrating phase composites with spinodal topologies. Compos. Struct..

[14] Stratford K, Adhikari R, Pagonabarraga I, Desplat J-C, Cates ME (2005). Colloidal jamming at interfaces: A route to fluid-bicontinuous gels. Science.

[15] Seker E, Reed ML, Begley MR (2009). Nanoporous gold: Fabrication, characterization, and applications. Materials.

[16] Martina M, Subramanyam G, Weaver JC, Hutmacher DW, Morse DE (2005). Developing macroporous bicontinuous materials as scaffolds for tissue engineering. Biomaterials.

[17] Garcia AE, Wang CS, Sanderson RN, McDevitt KM, Zhang Y (2019). Scalable synthesis of gyroid-inspired freestanding three-dimensional graphene architectures. Nanoscale Adv..

[18] Amani H, Mostafavi E, Arzaghi H, Davaran S, Akbarzadeh A (2018). Three-dimensional graphene foams: synthesis, properties, biocompatibility, biodegradability, and applications in tissue engineering. ACS Biomater. Sci. Eng..

[19] Lewis G (2013). Properties of open-cell porous metals and alloys for orthopaedic applications. J. Mater. Sci..

[20] Mour M, Das D, Winkler T, Hoenig E, Mielke G (2010). Advances in porous biomaterials for dental and orthopaedic applications. Materials.

[21] Miao X, Sun D (2009). Graded/gradient porous biomaterials. Materials.

[22] Albrektsson T, Dahlin C, Jemt T, Sennerby L, Turri A (2014). Is marginal bone loss around oral implants the result of a provoked foreign body reaction?. Clin. Implant Dent. Relat. Res..

[23] Hu J-M, Wang B, Ji Y, Yang T, Cheng X (2017). Phase-field based multiscale modeling of heterogeneous solid electrolytes: Applications to nanoporous Li3PS4. ACS Appl. Mater. Interfaces.

[24] Ngô B-N, Roschning B, Albe K, Weissmüller J, Markmann J (2017). On the origin of the anomalous compliance of dealloying-derived nanoporous gold. Scripta Mater..

[25] Cahn JW (1961). On spinodal decomposition. Acta Metall..

[26] Zheng L, Kumar S, Kochmann DM (2021). Data-driven topology optimization of spinodoid metamaterials with seamlessly tunable anisotropy. Comput. Methods Appl. Mech. Eng..

[27] Kumar S, Tan S, Zheng L, Kochmann DM (2020). Inverse-designed spinodoid metamaterials. npj Comput. Mater..

[28] Soyarslan C, Bargmann S, Pradas M, Weissmüller J (2018). 3D stochastic bicontinuous microstructures: Generation, topology and elasticity. Acta Mater..

[29] Sun C-T, Vaidya RS (1996). Prediction of composite properties from a representative volume element. Compos. Sci. Technol..

[30] Omairey SL, Dunning PD, Sriramula S (2019). Development of an ABAQUS plugin tool for periodic RVE homogenisation. Eng. Comput..

[31] Fullwood DT, Niezgoda SR, Kalidindi SR (2008). Microstructure reconstructions from 2-point statistics using phase-recovery algorithms. Acta Mater..

[32] Lebensohn RA, Kanjarla AK, Eisenlohr P (2012). An elasto-viscoplastic formulation based on fast Fourier transforms for the prediction of micromechanical fields in polycrystalline materials. Int. J. Plast..

[33] Song P, Yang T, Ji Y, Wang Z, Yang Z (2017). A comparison of Fourier spectral iterative perturbation method and finite element method in solving phase-field equilibrium equations. Commun. Comput. Phys..

[34] Rawat W, Wang Z (2017). Deep convolutional neural networks for image classification: A comprehensive review. Neural Comput..

[35] A. Garcia-Garcia, S. Orts-Escolano, S. Oprea, V. Villena-Martinez, and J. Garcia-Rodriguez, "A review on deep learning techniques applied to semantic segmentation," *arXiv,* 2017.

[36] Guo Y, Liu Y, Georgiou T, Lew MS (2018). A review of semantic segmentation using deep neural networks. Int. J. Multimed. Inform. Retr..

[37] G. Du, K. Wang and S. Lian, "Vision-based robotic grasping from object localization, pose estimation, grasp detection to motion planning: A review," *arXiv,* 2019.

[38] Murata T, Fukami K, Fukagata K (2020). Nonlinear mode decomposition with convolutional neural networks for fluid dynamics. J. Fluid Mech..

[39] de Oca Zapiain DM, Stewart JA, Dingreville R (2021). Accelerating phase-field-based microstructure evolution predictions via surrogate models trained by machine learning methods. npj Comput. Mater..

[40] Wang Z, Yang W, Xiang L, Wang X, Zhao Y (2022). Multi-input convolutional network for ultrafast simulation of field evolvement. Patterns.

[41] Yang K, Cao Y, Zhang Y, Fan S, Tang M (2021). Self-supervised learning and prediction of microstructure evolution with convolutional recurrent neural networks. Patterns.

[42] Jiang Z, Chen W, Burkhart C (2013). Efficient 3D porous microstructure reconstruction via Gaussian random field and hybrid optimization. J. Microsc..

[43] Challis VJ, Roberts AP, Grotowski JF, Zhang LC, Sercombe TB (2010). Prototypes for bone implant scaffolds designed via topology optimization and manufactured by solid freeform fabrication. Adv. Eng. Mater..

[44] Moerman KM (2018). GIBBON: The geometry and image-based bioengineering add-on. J. Open Source Softw..

[45] Frost HM (1994). Wolff's Law and bone’s structural adaptations to mechanical usage: An overview for clinicians. Angle Orthod..

[46] Wally ZJ, Haque AM, Feteira A, Claeyssens F, Goodall R (2019). Selective laser melting processed Ti6Al4V lattices with graded porosities for dental applications. J. Mech. Behav. Biomed. Mater..

[47] Marin E, Fusi S, Pressacco M, Paussa L, Fedrizzi L (2010). Characterization of cellular solids in Ti6Al4V for orthopaedic implant applications: Trabecular titanium. J. Mech. Behav. Biomed. Mater..

[48] Kladovasilakis N, Tsongas K, Tzetzis D (2020). Finite element analysis of orthopedic hip implant with functionally graded bioinspired lattice structures. Biomimetics.

[49] Vidyasagar A, Krödel S, Kochmann DM (2018). Microstructural patterns with tunable mechanical anisotropy obtained by simulating anisotropic spinodal decomposition. Proc. R. Soc. A.

[50] Nie Z, Jiang H, Kara LB (2019). Stress field prediction in cantilevered structures using convolutional neural networks. J. Comput. Inform. Sci. Eng..

[51] Bhatnagar S, Afshar Y, Pan S, Duraisamy K, Kaushik S (2019). Prediction of aerodynamic flow fields using convolutional neural networks. Comput. Mech..

[52] Karniadakis GE, Kevrekidis IG, Lu L, Perdikaris P, Wang S (2021). Physics-informed machine learning. Nat. Rev. Phys..

[53] Senhora FV, Sanders ED, Paulino GH (2022). Optimally-tailored spinodal architected materials for multiscale design and manufacturing. Adv. Mater..

[54] Ronneberger O, Fischer P, Brox T, Ronneberger O, Fischer P, Brox T (2015). U-net: Convolutional networks for biomedical image segmentation. International Conference on Medical image computing and computer-assisted intervention.

[55] Yang W, Wang Z, Yang T, He L, Song X (2021). Exploration of the underlying space in microscopic images via deep learning for additively manufactured piezoceramics. ACS Appl. Mater. Interfaces.

[56] Gostick JT, Khan ZA, Tranter TG, Kok MD, Agnaou M (2019). PoreSpy: A python toolkit for quantitative analysis of porous media images. J. Open Source Softw..

[57] Madadelahi M, Shamloo A, Salehi SS (2017). Numerical simulation of bio-chemical diffusion in bone scaffolds. Int. J. Med. Health Sci..

[58] Healy D, Timms NE, Pearce MA (2020). The variation and visualisation of elastic anisotropy in rock-forming minerals. Solid Earth.

