# Supplementary material for: Machine learning unifies flexibility and efficiency of spinodal structure generation for stochastic biomaterial design
Source: Sci Rep. 2023 Apr 3;13:5414. doi: 10.1038/s41598-023-31677-7 (PMC10070414; doi:10.1038/s41598-023-31677-7)
Supplement: Supplementary file 1 — Supplementary Information. [file 41598_2023_31677_MOESM1_ESM.docx]

*Supplementary Information*

**Machine learning unifies flexibility and efficiency of spinodal structure generation for stochastic biomaterial design**

Zhuo Wang^1^, Rana Dabaja^1^, Lei Chen^2,*^, Mihaela Banu^1,*^

*^1^ Department of Mechanical Engineering, University of Michigan, Ann Arbor, MI 48109, USA*

*^2^ Department of Mechanical Engineering, University of Michigan-Dearborn, Dearborn, MI 48128, USA*

*^*^ Corresponding authors*


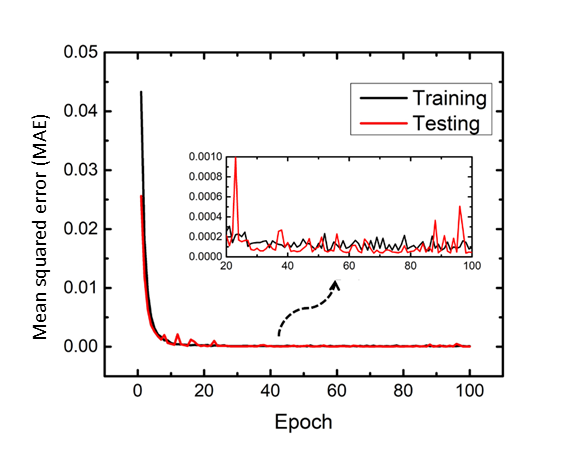


Supplementary Figure 1 Learning curve during training 3D CNN for modeling spinodal structure evolution.


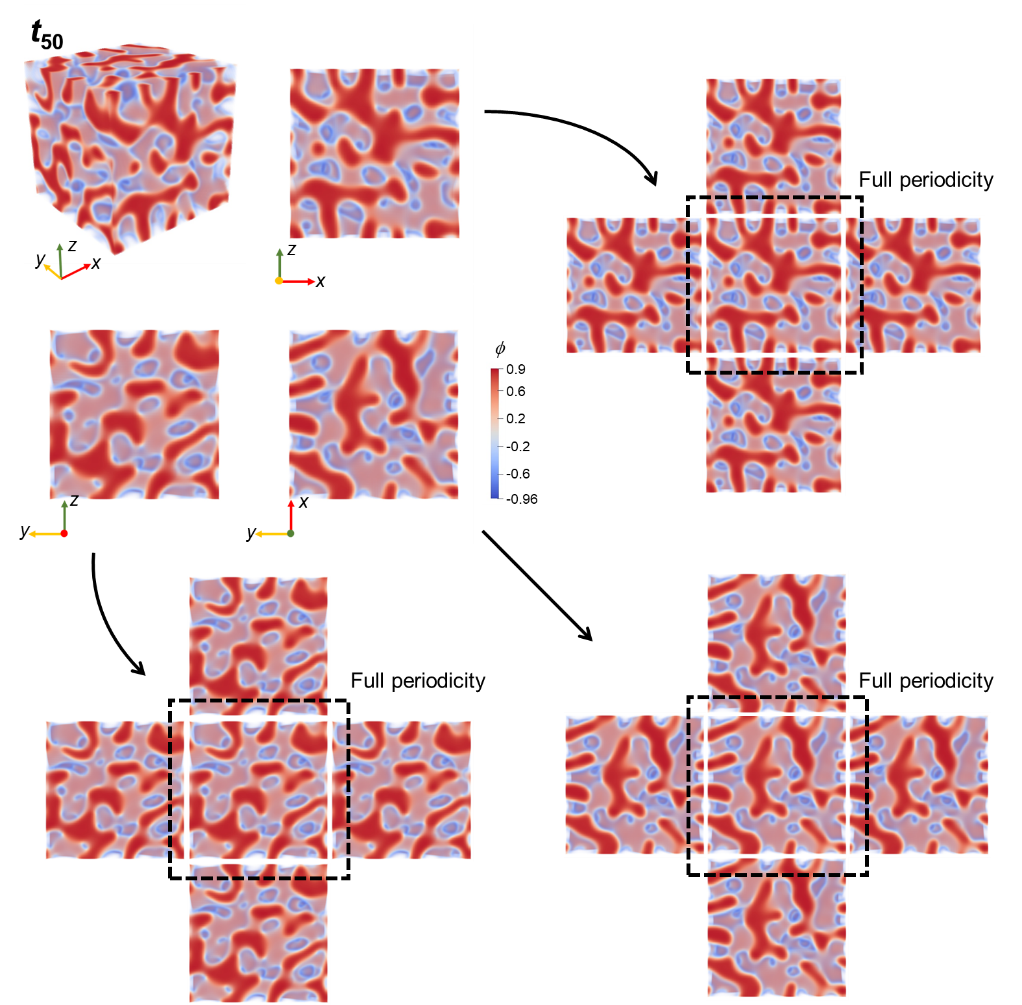


Supplementary Figure 2 3D periodicity of phase field result for step No. 50 of CNN based spinodal decomposition simulation. The cyclic padding successfully imposed periodic boundary condition for CNN-based simulation.


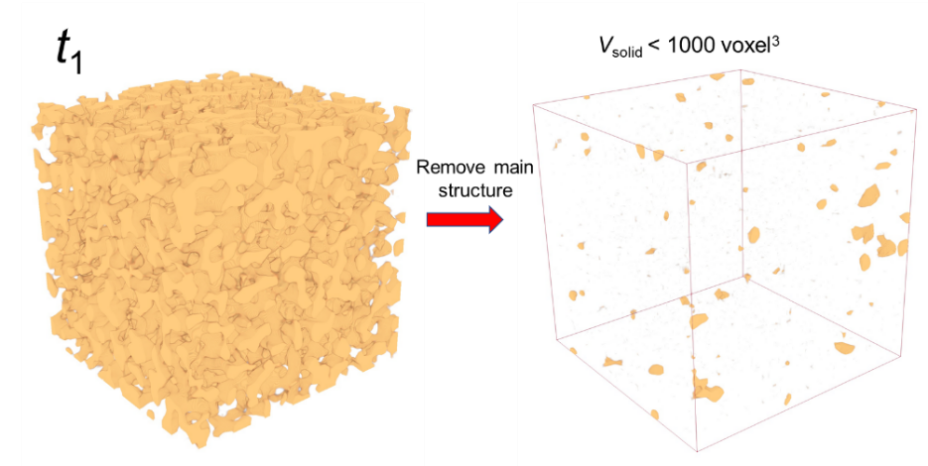


Supplementary Figure 3 Disjoint particles formed due to periodic boundary for porous structure derived at the early stage of spinodal decomposition (here step 1 for illustration purpose). The main spinodal structure, albeit with extreme randomness, has strict self-connectivity in 3D space.


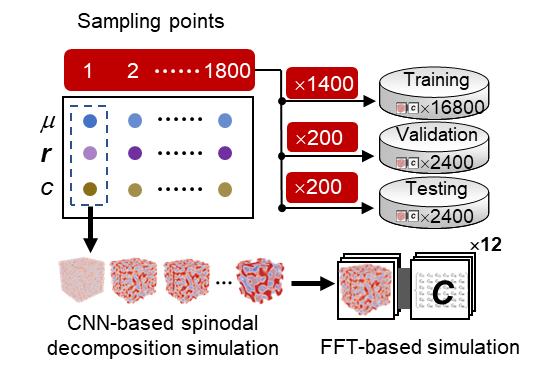


Supplementary Figure 4 Workflow of training and testing CNN for predicting elastic stiffness of anisotropic spinodal structure.


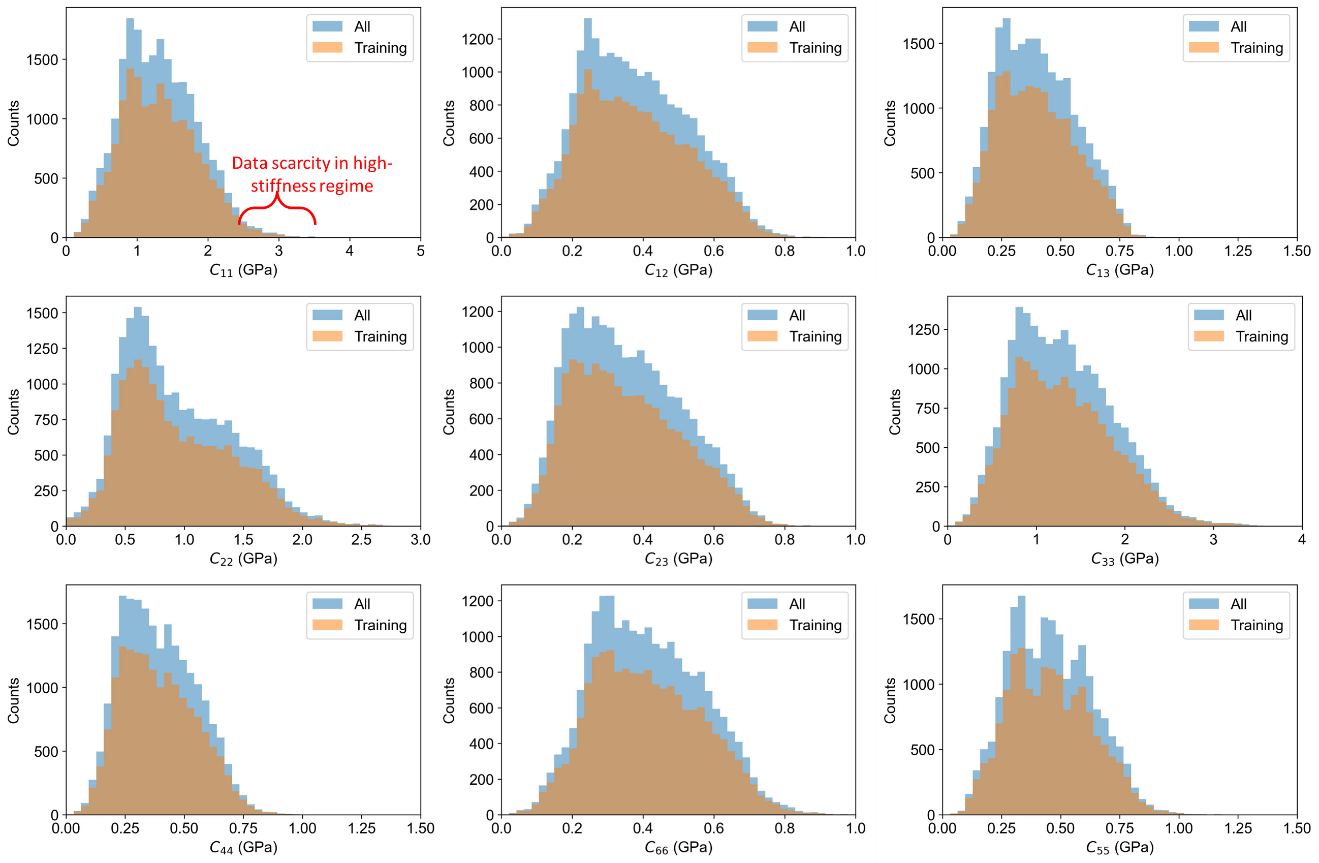


Supplementary Figure 5 Data distribution of the generated property dataset for training property prediction CNN.


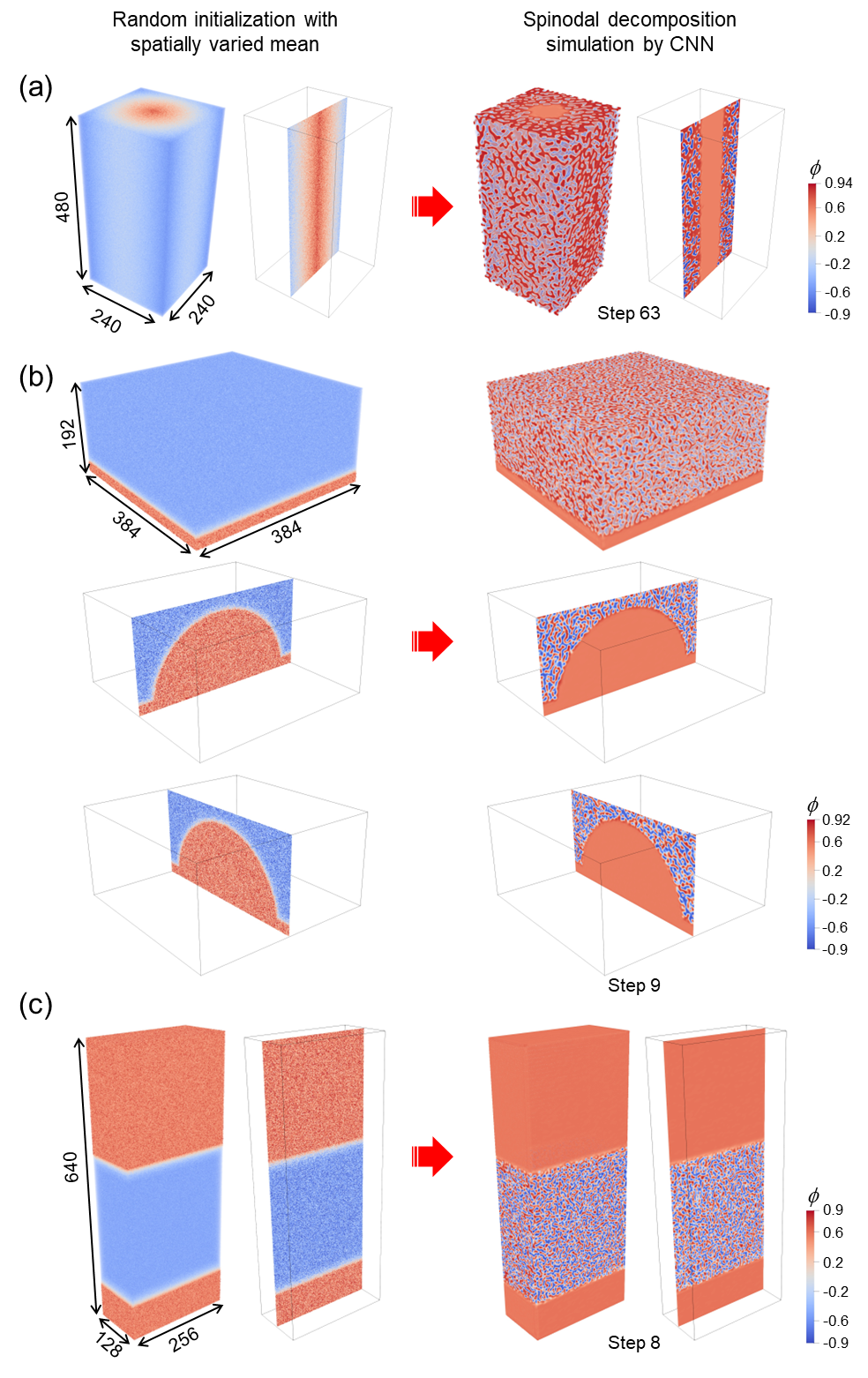


Supplementary Figure 6 Large-scale spinodal decomposition simulation enabled by CNN to obtain different gradient spinodal structures.


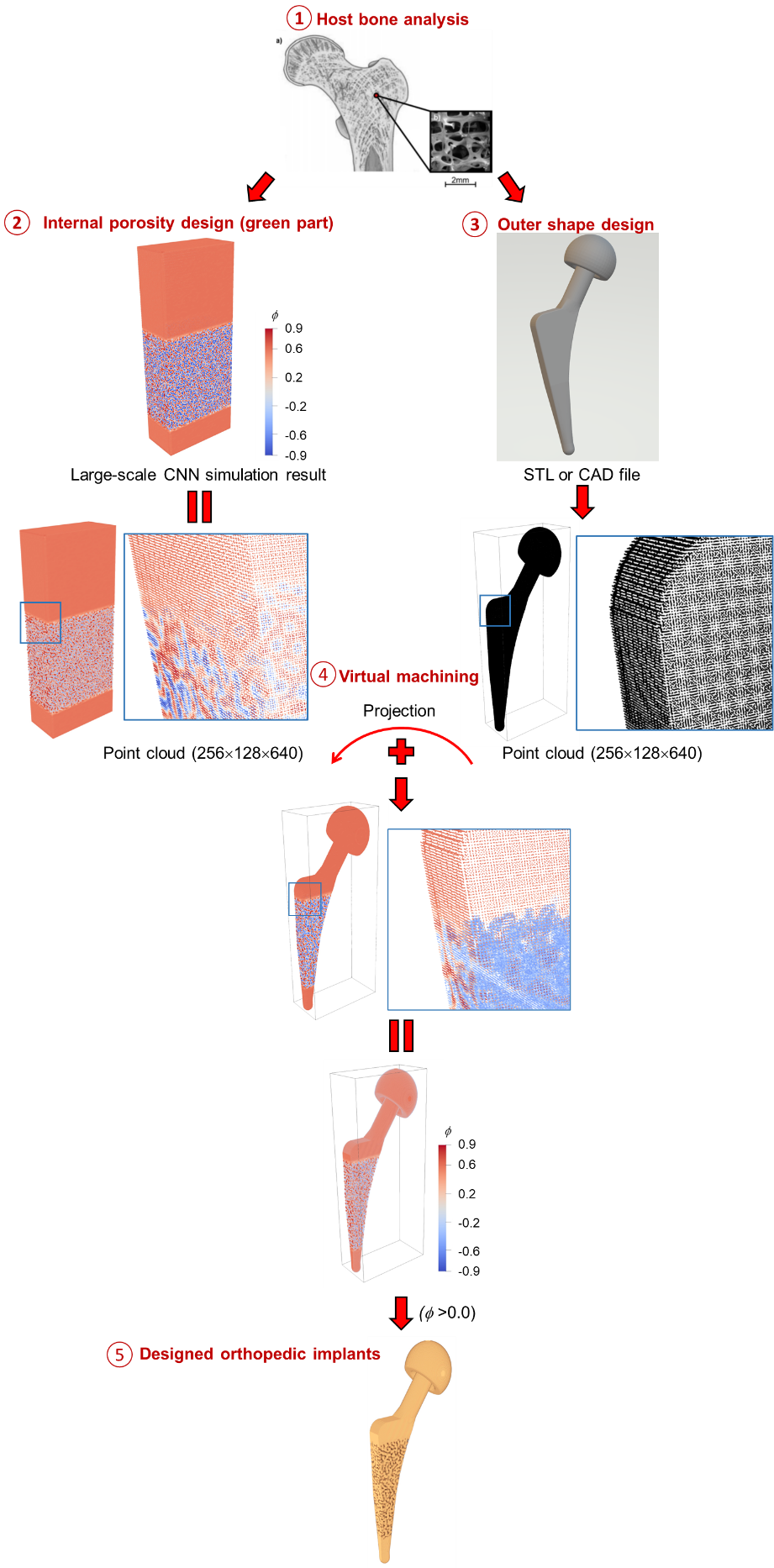


Supplementary Figure 7 Five-step design workflow for designing orthopedic implant with desired outer shape and internal porosity.


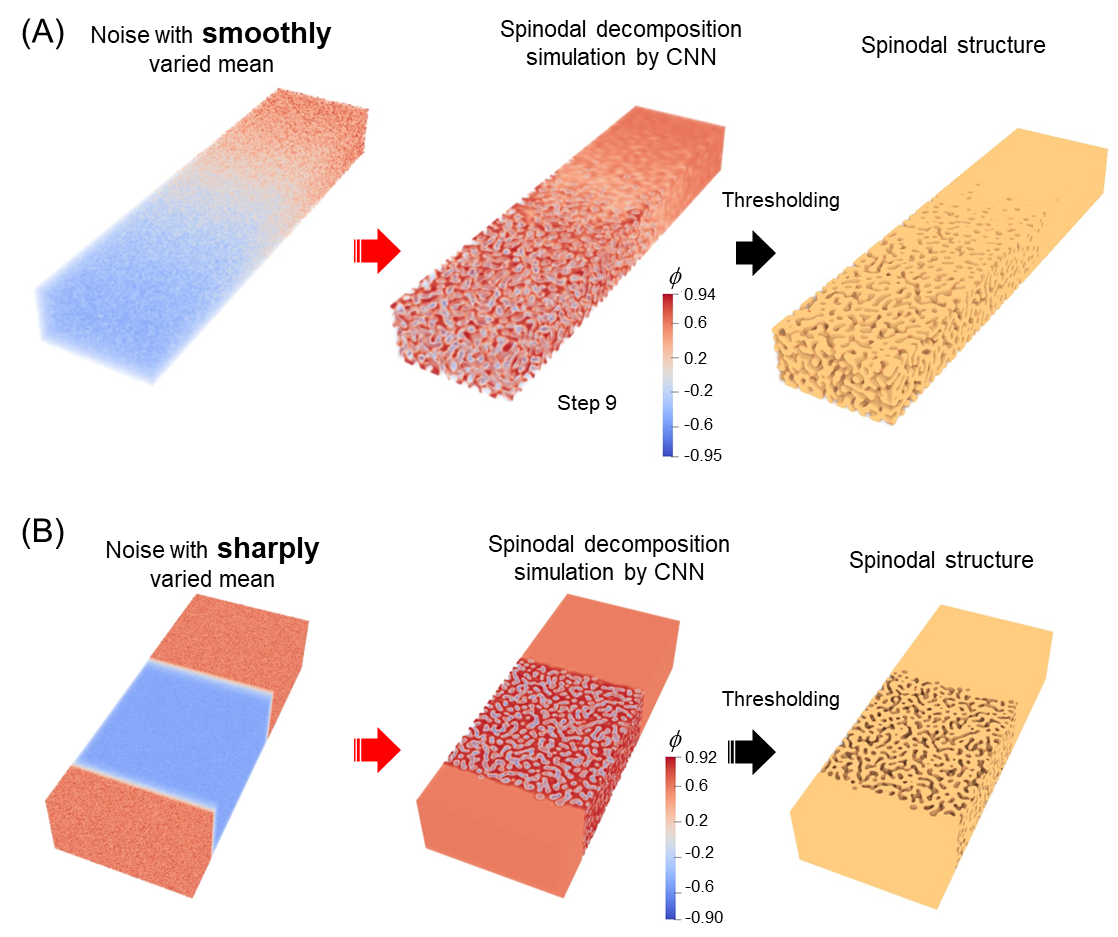


Supplementary Figure 8 By adjusting the spatially varied mean of noise input for CNN-based simulation, one can obtain gradient spinodal structure with (A) smooth solid-to-porous transition; (B) relatively harp solid-to-porous transition.


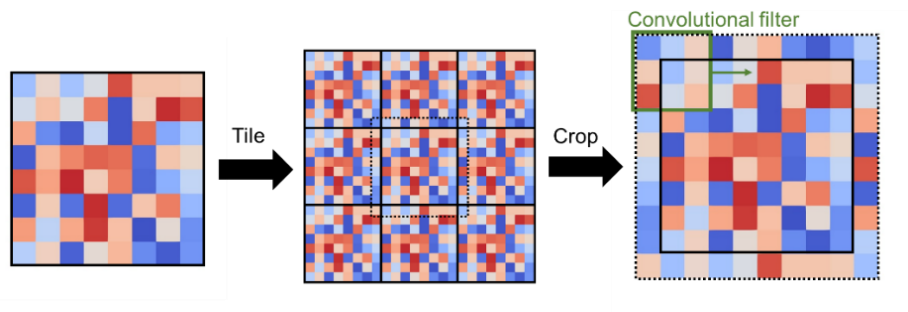


Supplementary Figure 9 Cyclic or periodic padding used for imposing periodic boundary in the current CNN. For illustration purpose, 2D case is shown.


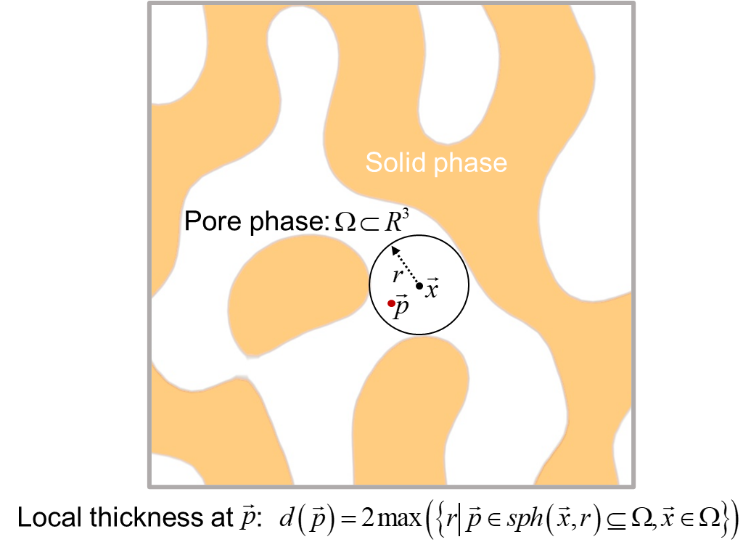


Supplementary Figure 10 Illustration of local thickness for calculating local pore size. The local thickness is equal to the diameter of the largest sphere that fits inside the pore channel and contains the point [1].


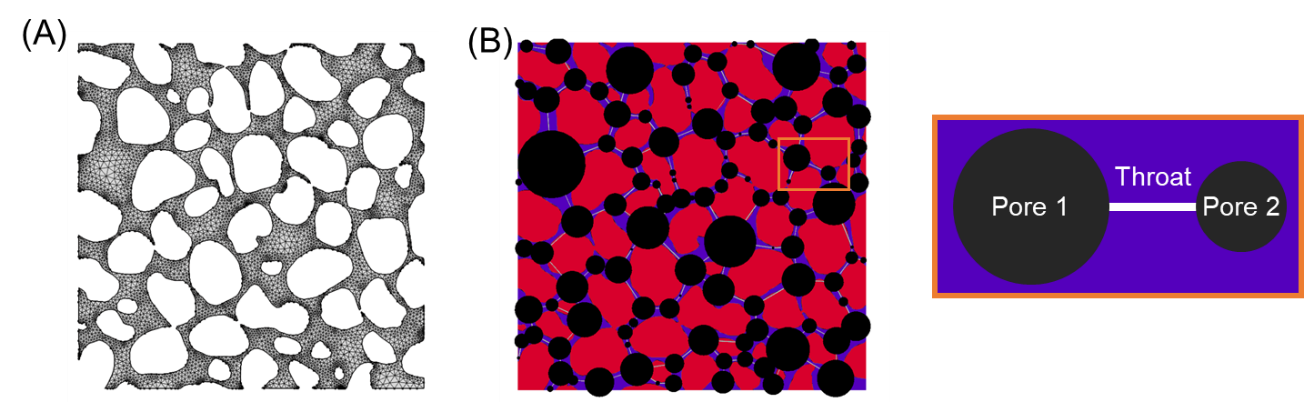


Supplementary Figure 11 (A) FEM meshing that resolves the detailed morphology and (B) pore network that represents the porous structure as a network of pipes [2].


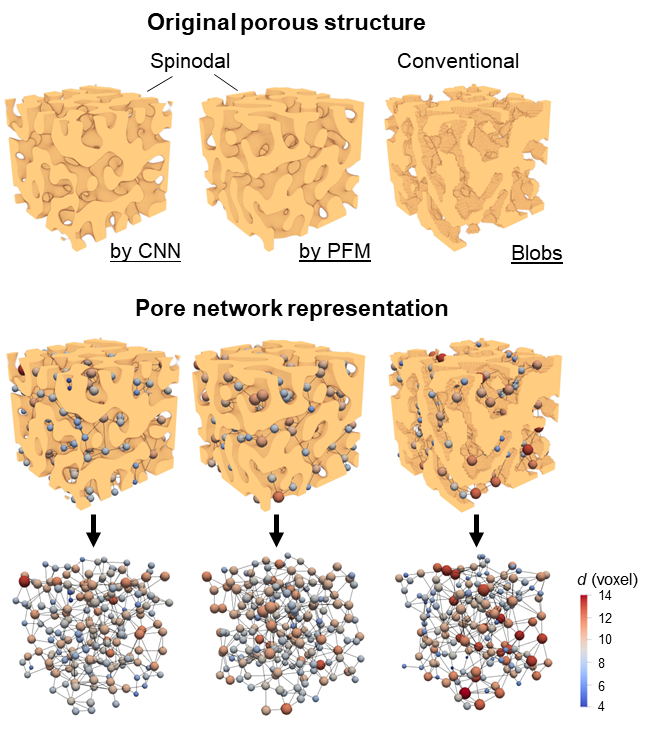


Supplementary Figure 12 Pore network representation of the studied porous structures for pore network modeling of diffusion.


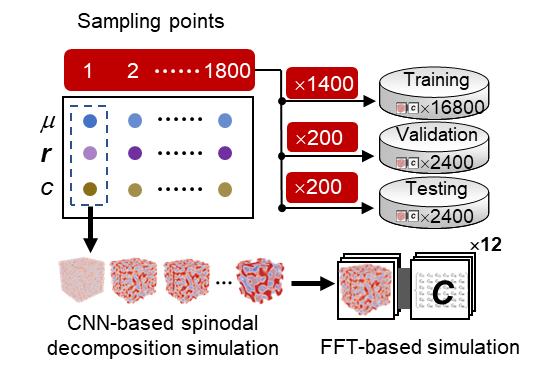


Supplementary Figure 13 Workflow of training and testing CNN for predicting elastic stiffness of anisotropic spinodal structure.


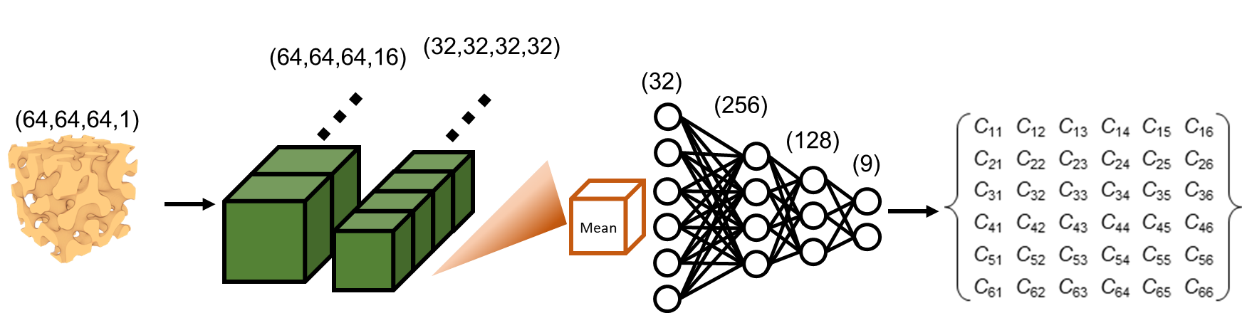


Supplementary Figure 14 Architecture of the adopted CNN for building linkage between 3D spinodal structure and its elastic stiffness, ***C***.


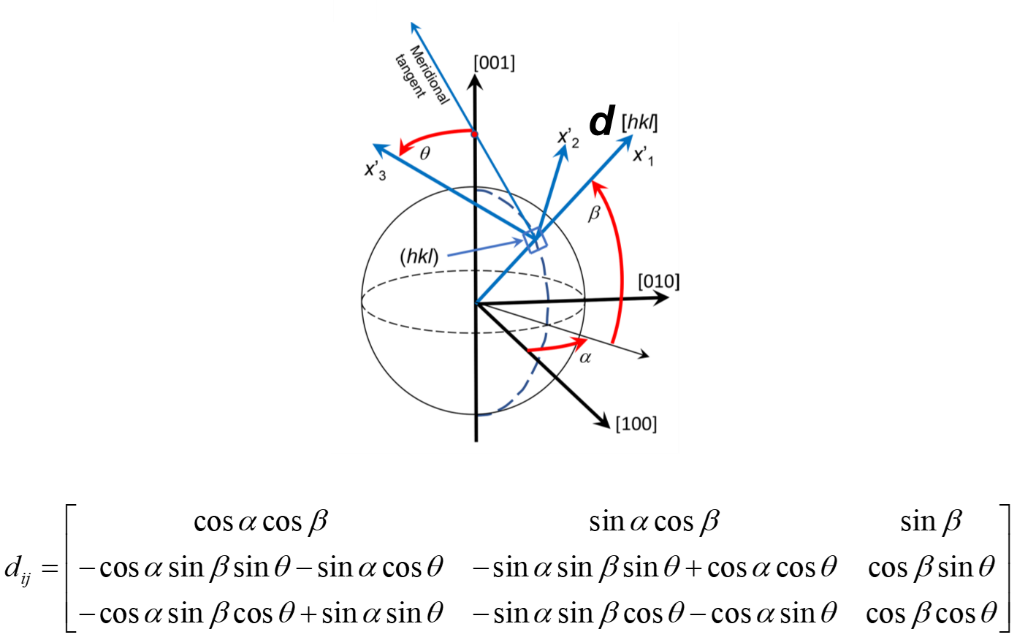


Supplementary Figure 15 Illustration of direction vector, ***d***, for calculating 3D direction-dependent Young’s modulus. (Image adapted from [3]).

Supplementary Table 1 Detailed architecture of the adopted 3D CNN for spinodal decomposition simulation

| **Layer name** | | **Output shape** |
| --- | --- | --- |
| Convolutional | Input | (64 , 64 , 64, 1) |
|  | Block1_conv1 | (64, 64, 64, 16) |
|  | Block1_conv2 | (64, 64, 64, 16) |
|  | Block2_conv1 | (64, 64, 64, 32) |
|  | Block2_conv2 | (64, 64, 64, 32) |
|  | Block3_conv1 | (64, 64, 64, 64) |
|  | Block3_conv2 | (64, 64, 64, 64) |
|  | Block4_conv1 | (64, 64, 64, 128) |
|  | Block4_conv2 | (64, 64, 64, 128) |
|  | Block5_conv1 | (64, 64, 64, 256) |
|  | Block5_conv2 | (64, 64, 64, 256) |
| Deconvolutional | Block6_conv1 | (64, 64, 64, 128) |
|  | Concatenate1 | (64, 64, 64, 256) |
|  | Block6_conv2 | (64, 64, 64, 128) |
|  | Block6_conv3 | (64, 64, 64, 128) |
|  | Block7_conv1 | (64, 64, 64, 64) |
|  | Concatenate2 | (64, 64, 64, 128) |
|  | Block7_conv2 | (64, 64, 64, 64) |
|  | Block7_conv3 | (64, 64, 64, 64) |
|  | Block8_conv1 | (64, 64, 64, 32) |
|  | Concatenate3 | (64, 64, 64, 64) |
|  | Block8_conv2 | (64, 64, 64, 32) |
|  | Block8_conv3 | (64, 64, 64, 32) |
|  | Block9_conv1 | (64, 64, 64, 16) |
|  | Concatenate4 | (64, 64, 64, 32) |
|  | Block9_conv2 | (64, 64, 64, 16) |
|  | Block9_conv3 | (64, 64, 64, 16) |
|  | Block10_conv1 | (64 , 64 , 64, 2) |
|  | Output | (64 , 64 , 64, 1) |

Supplementary Table 2 Detailed architecture of the adopted 3D CNN for elastic property prediction

| **Layer name** | | **Output shape** |
| --- | --- | --- |
| Encoder | Input | (64 , 64 , 64, 1) |
|  | Block1_conv1 | (64, 64, 64, 16) |
|  | Block1_conv2 | (64, 64, 64, 16) |
|  | Block2_conv1 | (32, 32, 32, 32) |
|  | Block2_conv2 | (32, 32, 32, 32) |
|  | Average pooling | (32) |
| Fully connected layer | Dense_1 | (256) |
|  | Dense_2 | (128) |
|  | Dense_3 | (64) |
|  | Output | (9) |

**References**

[1] M. D. Abràmoff, P. J. Magalhães and S. J. Ram, "Image processing with ImageJ," *Biophotonics international,* vol. 11, pp. 36-42, 2004.

[2] J. Gostick, M. Aghighi, J. Hinebaugh, T. Tranter, M. A. Hoeh*, et al.*, "OpenPNM: a pore network modeling package," *Computing in Science & Engineering,* vol. 18, pp. 60-74, 2016.

[3] D. Healy, N. E. Timms and M. A. Pearce, "The variation and visualisation of elastic anisotropy in rock-forming minerals," *Solid earth,* vol. 11, pp. 259-286, 2020.
